# Association of improved oxidative stress tolerance and alleviation of glucose repression with superior xylose-utilization capability by a natural isolate of *Saccharomyces cerevisiae*

**DOI:** 10.1186/s13068-018-1018-y

**Published:** 2018-02-05

**Authors:** Cheng Cheng, Rui-Qi Tang, Liang Xiong, Ronald E. Hector, Feng-Wu Bai, Xin-Qing Zhao

**Affiliations:** 1 State Key Laboratory of Microbial Metabolism, School of Life Sciences and Biotechnology, Shanghai Jiao Tong University, Shanghai, 200240 China; 2 School of Life Science and Biotechnology, Dalian University of Technology, Dalian, 116024 China; 3 Bioenergy Research Unit, National Center for Agricultural Utilization Research, USDA-ARS, Peoria, IL USA

**Keywords:** *Saccharomyces cerevisiae*, Transcriptomic analysis, Xylose utilization, Oxidative stress, Transcription factor, Glucose repression

## Abstract

**Background:**

*Saccharomyces cerevisiae* wild strains generally have poor xylose-utilization capability, which is a major barrier for efficient bioconversion of lignocellulosic biomass. Laboratory adaption is commonly used to enhance xylose utilization of recombinant *S. cerevisiae*. Apparently, yeast cells could remodel the metabolic network for xylose metabolism. However, it still remains unclear why natural isolates of *S. cerevisiae* poorly utilize xylose. Here, we analyzed a unique *S. cerevisiae* natural isolate YB-2625 which has superior xylose metabolism capability in the presence of mixed-sugar. Comparative transcriptomic analysis was performed using *S. cerevisiae* YB-2625 grown in a mixture of glucose and xylose, and the model yeast strain S288C served as a control. Global gene transcription was compared at both the early mixed-sugar utilization stage and the latter xylose-utilization stage.

**Results:**

Genes involved in endogenous xylose-assimilation (*XYL2* and *XKS1*), gluconeogenesis, and TCA cycle showed higher transcription levels in *S. cerevisiae* YB-2625 at the xylose-utilization stage, when compared to the reference strain. On the other hand, transcription factor encoding genes involved in regulation of glucose repression (*MIG1*, *MIG2*, and *MIG3*) as well as *HXK2* displayed decreased transcriptional levels in YB-2625, suggesting the alleviation of glucose repression of *S. cerevisiae* YB-2625. Notably, genes encoding antioxidant enzymes (*CTT1*, *CTA1*, *SOD2,* and *PRX1*) showed higher transcription levels in *S. cerevisiae* YB-2625 in the xylose-utilization stage than that of the reference strain. Consistently, catalase activity of YB-2625 was 1.9-fold higher than that of *S. cerevisiae* S288C during the xylose-utilization stage. As a result, intracellular reactive oxygen species levels of *S. cerevisiae* YB-2625 were 43.3 and 58.6% lower than that of S288C at both sugar utilization stages. Overexpression of *CTT1* and *PRX1* in the recombinant strain *S. cerevisiae* YRH396 deriving from *S. cerevisiae* YB-2625 increased cell growth when xylose was used as the sole carbon source, leading to 13.5 and 18.1%, respectively, more xylose consumption.

**Conclusions:**

Enhanced oxidative stress tolerance and relief of glucose repression are proposed to be two major mechanisms for superior xylose utilization by *S. cerevisiae* YB-2625. The present study provides insights into the innate regulatory mechanisms underlying xylose utilization in wild-type *S. cerevisiae*, which benefits the rapid development of robust yeast strains for lignocellulosic biorefineries.

**Electronic supplementary material:**

The online version of this article (10.1186/s13068-018-1018-y) contains supplementary material, which is available to authorized users.

## Background

Bioconversion of renewable lignocellulosic biomass to biofuels and biochemicals is environment-friendly and sustainable. Budding yeast *Saccharomyces cerevisiae* has been widely studied to produce cellulosic ethanol. However, bioconversion of lignocellulosic biomass is still not economically feasible. One major bottleneck is that most natural *S. cerevisiae* strains cannot efficiently use xylose, which is abundant in lignocellulosic hydrolysates. Therefore, multiple genetic engineering strategies have been adopted to enable xylose utilization in *S. cerevisiae* [1–3].

It is well known that genetic background of the *S. cerevisiae* strain exerts significant effects on the performance of recombinant xylose-assimilating strains. Expression of the same set of genes in different *S. cerevisiae* strains has resulted in variable xylose utilization abilities [4–6]. Although screening of suitable host strains is commonly employed to construct engineered yeasts, the process is labor-intensive and time-consuming. On the other hand, evolutionary engineering of the recombinant strains is always required to achieve satisfactory xylose-utilization performance [1, 7]. Unveiling the underlying mechanisms of host dependence is beneficial to promote efficient and rapid strain development.

It was revealed that multiple gene mutations occur during adaptive evolution, and some key genes leading to improved xylose utilization have been identified, such as *PHO13* and *ASK10* [8, 9]. It is very important to explore the innate regulatory mechanisms in the host strains, which are responsible for the optimized metabolic flux for xylose utilization. However, related studies have been only performed using the recombinant strains or the evolved recombinants [4, 9–11].

Natural isolates of *S. cerevisiae* are rich sources of robust hosts for genetic engineering of xylose utilization. Currently, most studies on natural yeast isolates focused on the differences of stress tolerance [12]. In contrast, so far, limited study has been performed on the xylose-utilization properties of natural *S. cerevisiae* isolates. Despite the common knowledge that natural *S. cerevisiae* strains cannot use xylose as the sole carbon source, it was reported that some natural *S. cerevisiae* strains, especially wine strains, could grow weakly in xylose [13, 14]. In addition, key endogenous genes responsible for xylose utilization in the wild *S. cerevisiae* strains were identified [14, 15].

We are interested in whether more native *S. cerevisiae* strains that can use xylose can be explored, and have screened wild yeast isolates and compared their xylose-assimilation capability. We found that *S. cerevisiae* YB-2625 isolated from bagasse showed superior xylose consumption capability among all the *S. cerevisiae* wild strains when tested in mixture of glucose and xylose. Consistently, it was reported that comparing the engineered yeasts with other hosts, *S. cerevisiae* YRH396 derived from *S. cerevisiae* YB-2625 showed more biomass accumulation and faster growth rate using xylose as the sole carbon source [5]. It is of significance to explore the molecular mechanisms underlying this unique xylose-utilization performance, and therefore, in this study, *S. cerevisiae* YB-2625 was selected for comparative transcriptomic analysis. The model yeast strain S288C was chosen as the reference strain [16].

The differences of global gene transcription among the recombinant yeast strains have been studied using xylose or glucose as the sole carbon source [4, 17, 18]. However, we investigated mixed-sugar utilization in this study, because both glucose and xylose are present in the cellulosic hydrolysate. Two different physiological stages, including the log growth phase for mixed-sugar utilization and the early xylose-utilization stage which is initiated after complete consumption of glucose, were investigated. To our best knowledge, this is the first report about the transcriptomic analysis of a natural *S. cerevisiae* isolate in the condition of mixed-sugar (glucose and xylose) fermentation. Our results provide novel insights into understanding the impact of the host selection and will contribute to identifying useful genetic targets for improving xylose utilization of the recombinant yeasts.

## Methods

### Yeast strains, media, and culture conditions

*Saccharomyces cerevisiae* YB-2625 was obtained from Agricultural Research Service (ARS) Culture Collection, US Department of Agriculture (USDA), USA. The reference yeast S288C (ATCC 204508) was preserved in our lab. Xylose-fermenting recombinant YRH396 was constructed previously using *S. cerevisiae* YB-2625 as the host strain [5]. Yeast strains were stored in 30% glycerol at − 80 °C. For seed culture, cells were cultivated in YPD medium containing 10 g/L yeast exact, 20 g/L peptone, and 20 g/L glucose to stationary phase, and then transferred into fresh YPD medium overnight. The mixed-sugar fermentation medium (YPD80X20) consists of 4 g/L yeast exact, 3 g/L peptone, 80 g/L glucose, and 20 g/L xylose, while 40 g/L xylose (YPX40) was used as sole carbon source when performing xylose fermentation. After seed culture, the cells were inoculated into 100 mL YPD80X20 or YPX40 medium in 250 mL flasks with the initial OD_600_ 0.2 at 30 °C, 150 rpm under micro-aerobic condition.

### Transcriptome analysis and real-time quantitative PCR (RT-qPCR) analysis

Samples were taken at the stages of mixed-sugar fermentation (~ 7 h after inoculation) and xylose fermentation (~ 48 h), respectively. Cell pellets were collected by centrifugation at 8000×*g* for 5 min and then immediately frozen in liquid nitrogen. Total RNA was extracted by Spin Column Plant total RNA Purification Kit (Sangon, Shanghai, China) according to the manufacturer’s instructions. Two independently replicated experiments of mixed-sugar fermentation and RNA-seq analysis were performed. Agilent 2100 Bioanalyzer was used for determining RNA quality and quantity, and the RNA integrity number (RIN) of all the samples was more than 9.5. The RNA-seq libraries were sequenced on IlluminaHiseq 4000 and analyzed by Beijing Genomics Institute (BGI, Shenzhen, China). All the fold changes of transcriptomic data in this work are log 2 ratios. Five genes involved in stress response, xylose-utilization pathway, and ethanol formation pathway were selected for RT-qPCR verification of the RNA-seq analysis results. The primers used in this study are all listed in Additional file 1: Table S1. The RT-qPCR analysis was performed following the manufacturer’s protocol of SYBR^®^ Premix Ex Taq™ II (Takara Kyoto, Japan), and *ACT1* was selected as an internal control. The relative expression level of genes was determined by the 2^−ΔΔCt^ method [19].

### Quantification of reactive oxygen species (ROS)

ROS accumulation in yeast cells taken in 7 h and 48 h during mixed-sugar fermentation was immediately detected using the ROS detection kit (Beyotime Institute of Biotechnology, Shanghai, China). OD_600_ values of the two yeast strains (YB-2625 and S288C) were adjusted to the same level by PBS buffer (pH 7.3–7.5) and then determined using the oxidant-sensitive probe 2′,7′-dichlorofluorescin diacetate (DCFH-DA) as described elsewhere [20].

### CAT activity determination

Crude protein was extracted from yeast cells collected at 7 and 48 h of mixed-sugar fermentation as previously described [21], and was subsequently subjected to the detection of catalase (CAT) activity. The activity of CAT was determined with a reagent kit according to the manufacturer’s instructions (Beyotime Institute of Biotechnology, Shanghai, China).

### Oxidative stress tolerance assay

Yeast strains were cultured in YPD medium for 24 h at 30 °C, 150 rpm, and then transferred to fresh YPD medium and cultivated until stationary stage. Before spot assay, the cultures were adjusted to the same initial cell concentration (OD_600_ ~ 1.0) and serially diluted by tenfold. Two-microliter suspension from each dilution was spotted on YPD plates containing 5 mM hydrogen peroxide (H_2_O_2_) and incubated at 30 °C. To minimize degradation of H_2_O_2_ in the plates, H_2_O_2_ was added to the pre-cooled YPD medium and mixed well immediately prior to pouring. Experiments were performed in triplicates and the YPD plates without H_2_O_2_ served as control.

### Plasmids and strains construction

The *CTT1* and *PRX1* genes were amplified from the genomic DNA of YB-2625 using primer pairs *CTT1*-F’/*CTT1*-R’ and *PRX1*-F’/*PRX1*-R’ as listed in Additional file 1: Table S1. The fragments were digested by *Hind*III and *Pac*Ι, and then cloned into plasmid pRS41H-*PGK1*_*p*_, which contains a *PGK1* promoter and *CYC1* terminator, to create pRS41H-*CTT1* and pRS41H-*PRX1*. The plasmids were transformed into YRH396 using the lithium acetate method [22]. Cells were selected on agar plates with YPD and 300 μg/mL hygromycin. The transformants were confirmed by PCR verification using *CTT1*-F’ or *PRX1*-F’ and *hph*-in-R’.

### Total ergosterol content determination

Cells from 10 mL of fermentation broth under the same condition of transcriptomic analysis were collected at 10,000×*g* for 3 min and then washed with sterilized distilled water twice. Ergosterol was extracted and detected as described in the previous study [23].

### Cell growth evaluation and HPLC analysis

Cell growth was determined by optical density at 600 nm (OD_600_). Samples from fermentation broth were analyzed by HPLC (Waters 410, Waters, MA, USA) equipped with an Aminex HPX-87H column (300 mm × 7.8 mm, Bio-Rad, Hercules, CA) as previously described to determine concentrations of glucose, xylose, ethanol, glycerol and acetic acid [24].

### Statistical analysis

All quantitative data were expressed as the mean value with corresponding standard deviation (SD) obtained from three independent experiments. The statistical analysis showed in enzyme detection section was performed using Student’s *t* test at the significance of *p* < 0.05 and *p* < 0.01, respectively.

## Results and discussion

### Xylose-utilization performance of *S. cerevisiae* YB-2625 and *S. cerevisiae* S288C

**Fig. 1:**
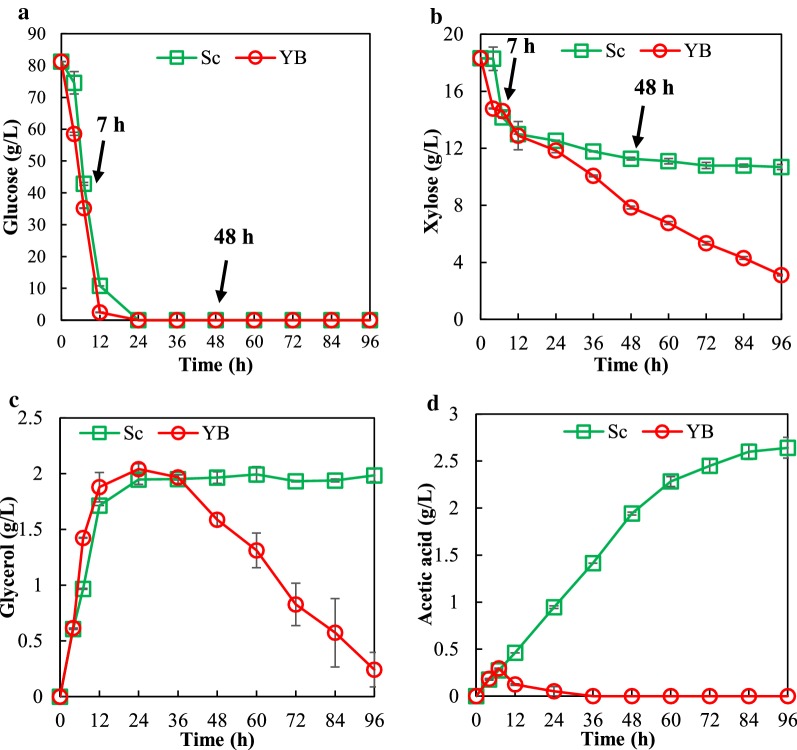
Fermentation performance of *S. cerevisiae* YB-2625 (YB) and *S. cerevisiae* S288C (Sc) in the presence of mixed sugar. Batch fermentation was performed in 100 mL medium containing 4 g/L yeast extract, 3 g/L peptone, 80 g/L glucose, and 20 g/L xylose in 250 mL Erlenmeyer flasks with initial OD_600_ ~ 0.2 at 150 rpm and 30 °C under micro-aerobic condition. **a** and **b** glucose and xylose consumption; **c** and **d** glycerol and acetic acid generation. The green and red colors stand for *S. cerevisiae* S288C and *S. cerevisiae* YB-2625, respectively. The arrows indicate the time points for comparative transcriptomic analysis (i.e. 7 and 48 h). The results shown were the mean values of triplicate experiments

When evaluating mixed-sugar fermentation performance of *S. cerevisiae* YB-2625 (shortened as YB-2625 in the following text) and the control strain *S. cerevisiae* S288C (shortened as S288C) in YPD80X20 medium, YB-2625 showed a higher growth rate than S288C throughout the culture process (Additional file 1: Figure S1A), and it also showed faster sugar utilization rate when compared to S288C. As shown in Fig. 1, a significant difference between the two strains in xylose utilization was observed. YB-2625 consumed 15.2 g/L xylose in 96 h, whereas S288C only consumed 7.6 g/L xylose. Correspondingly, 10.9 and 3.1 g/L of xylitol were detected in the fermentation media of YB-2625 and S288C, respectively, at 96 h, without any obvious difference in the ethanol concentration (Additional file 1: Figure S1B, C). Interestingly, accumulation of glycerol and acetic acid, the two main by-products in ethanol production, was rather different. Glycerol production by S288C was elevated throughout the fermentation process, but for YB-2625, the highest glycerol production appeared at 24 h. After that, the glycerol was co-consumed with xylose. As shown in Fig. 1c, the final concentration of glycerol produced by YB-2625 was 0.24 g/L, which was much lower when compared to 1.99 g/L glycerol produced by S288C. In addition, acetic acid production of S288C was 2.6 g/L in 96 h; however, there was no detectable acetic acid generated by YB-2625 (Fig. 1d). These results suggest that YB-2625 may be endowed with specific mechanisms of carbon metabolism that are lacking in S288C.

### Overview of the transcriptomic analysis data and validation by RT-qPCR

Samples of the two strains were collected from both mixed-sugar (xylose and glucose, XG) utilization phase and xylose (X) utilization phase, and the two comparative groups were named YBXG vs SCXG and YBX vs SCX, respectively. When comparing YBXG with SCXG, 1637 differentially expressed genes were observed, including 1313 downregulated and 324 upregulated genes as screening the value by Log2 ratio ≥ 2.0 and FDR ≤ 0.001. Meanwhile, there were 2004 differentially expressed genes in the comparative group YBX vs SCX, with 1727 downregulated and 277 upregulated genes (Additional file 1: Figure S1D). It is notable that most changed genes were downregulated in YB-2625. All the changed genes described in the text are listed in Additional file 1: Table S5.

To validate the reliability of the RNA-seq data, the transcription levels of five genes involved in stress response, xylose metabolism, and ethanol generation pathway were confirmed by RT-qPCR analysis. Consistent data were observed in the RT-qPCR results for all five genes (Additional file 1: Table S2), suggesting that the transcriptomic data are reliable.

#### Gene Ontology (GO) terms analysis

**Fig. 2:**
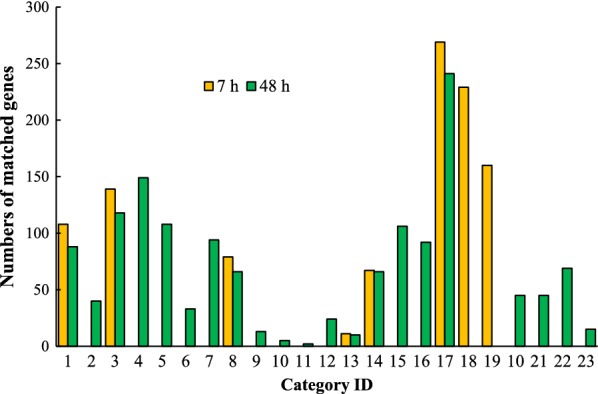
Go terms’ enrichment analysis of the changed genes during mixed-sugar fermentation by comparing *S. cerevisiae* YB2625 and *S. cerevisiae* S288C. Yeast cells were collected at the above-mentioned time points (Fig. 1) and the RNA samples prepared were subjected to RNA sequencing. The significantly changed genes (Log2 ratio ≥ 1.0 and FDR ≤ 0.001) were analyzed by the MIPS functional catalog database and screened by *p* value ≤ 0.05. MIPS FunCats and number of matched genes in the two sugar utilization stages. The orange and green bars stand for YBXG vs SCXG (7 h) and YBX vs SCX (48 h), respectively. Definitions of MIPS FunCats were listed in Table 1. YB and SC stand for *S. cerevisiae* YB-2625 and *S. cerevisiae* S288C, respectively. XG and X represent mixed sugar (xylose and glucose) and xylose, respectively

After analyzing the changed genes (Log2 ratio ≥ 1.0 and FDR ≤ 0.001) by the MIPS functional catalog database, categories were summarized and selected through screening by *p* value ≤ 0.05. GO term analysis showed that significantly changed genes were enriched in cell cycle, nucleotide metabolism, DNA synthesis, and replication, as well as vitamin and cofactor metabolism at both stages. More importantly, genes involved in alcohol fermentation were enriched in both two stages when comparing YB-2625 with S288C. Remarkably, several enrichments only presented in YB-2625 during the mixed-sugar fermentation when compared to S288C, which mainly included ribosome biogenesis and translation, indicating that at this stage, protein synthesis is more active in YB-2625 than S288C. However, during xylose utilization, the changes that occurred in transcription profiles were related to DNA modification and lipid metabolism. As shown in Fig. 2 and Table 1, the enrichment of genes involved in DNA damage response, detoxification, peroxisome as well as homeostasis were shown at 48 h when comparing YB-2625 with S288C, which suggested that stress response was more evident during the xylose-utilization stage. Our results are consistent with the previous report using recombinant *S. cerevisiae* that showed stress response was related to xylose utilization [4]. Changes in transcription levels of stress-related genes were also observed in the xylose-utilizing *S. cerevisiae* strains [14]. Interestingly, differences of gene transcription levels in phospholipid, phosphate, and sulfur and lipid metabolism were also observed during the xylose consumption stage (Fig. 2 and Table 1).

**Table 1:** Definitions of MIPS FunCats and number of matched genes in the two sugar utilization stages

ID	Functional category		7 h	*p* value	48 h	*p* value
1	01.01	Amino acid metabolism	108 (6.51%)	9.90e−03	88 (6.81%)	6.76e−03
2	01.02	Nitrogen, sulfur and selenium metabolism	0	7.39e−02	40 (3.09%)	4.30e−02
3	01.03	Nucleotide/nucleoside/nucleobase metabolism	139 (8.38%)	4.37e−03	118 (9.14%)	4.08e−04
4	01.04	Phosphate metabolism	0 (149)	1.00e+00	149 (11.5%)	6.56e−03
5	01.06	Lipid, fatty acid, and isoprenoid metabolism	0 (102)	1.00e+00	108 (8.36%)	1.21e−02
6	01.06.02	Membrane lipid metabolism	0 (25)	1.00e+00	33 (2.55%)	2.70e−02
7	01.06.02.01	Phospholipid metabolism	0 (22)	1.00e+00	94 (2.32%)	1.44e−02
8	01.07	Metabolism of vitamins, cofactors, and prosthetic groups	79 (4.76%)	3.78e−03	66 (5.11%)	1.66e−03
9	02.07	Pentose-phosphate pathway	0 (10)	3.19e−01	13 (1.00%)	6.71e−03
10	02.07.01	Pentose-phosphate pathway oxidative branch	0 (3)	2.20e−01	5 (0.38%)	2.42e−03
11	02.11.07	Regulation of electron transport and membrane-associated energy conservation	0 (1)	4.82e−01	2 (0.15%)	4.76e−02
12	02.16	Fermentation	0 (21)	7.78e−02	24 (1.85%)	3.21e−04
13	02.16.01	Alcohol fermentation	11 (0.66%)	5.94e−03	10 (0.77%)	3.11e−03
14	10.01.03	DNA synthesis and replication	67 (4.04%)	2.88e−02	66 (5.11%)	5.73e−05
15	10.01.05	DNA recombination and DNA repair	0 (111)	1.69e−01	106 (8.21%)	5.71e−04
16	10.01.09	DNA restriction or modification	0 (89)	1.00e+00	92 (7.12%)	9.23e−03
17	10.03	Cell cycle	269 (16.2%)	2.90e−02	241 (18.6%)	8.67e−06
18	12.01	Ribosome biogenesis	229 (13.8%)	3.28e−49	0 (53)	1.00e+00
19	12.04	Translation	160 (9.65%)	2.91e−30	0 (44)	1.00e+00
10	32.01.09	DNA damage response	0 (47)	3.97e−01	45 (3.48%)	3.78e−02
21	32.7	Detoxification	0 (50)	2.16e−01	45 (3.48%)	3.78e−02
22	34.01	Homeostasis	0 (65)	1.00e+00	69 (5.34%)	3.49e−02
23	70.19	Peroxisome	0 (9)	1.00e+00	15 (1.16%)	2.74e−02

The percentage was calculated by classified genes/all the significantly changed genes

7 h, glucose utilization stage; 48 h, xylose utilization stage

#### Enrichment of transcription factors that putatively regulate the differentially transcribed genes

**Fig. 3:**
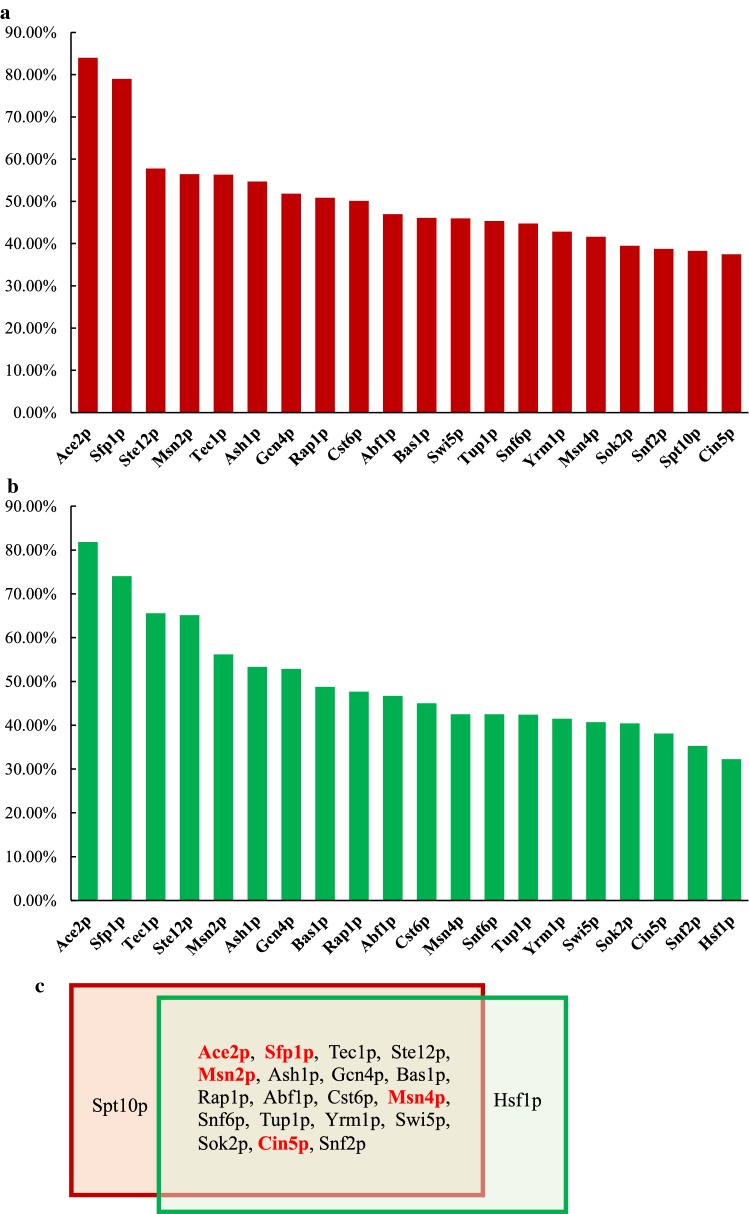
Top 20 enriched transcription factors (TFs) in the two sugar utilization conditions. **a** Enriched TFs in the condition of YBXG vs SCXG; **b** enriched TFs in the condition of YBX vs SCX; **c** common and specific TFs of the top 20 enriched transcription factors in the two conditions (XG and X, respectively). The TFs marked in red mean that the genes are related to stress response

Potential transcription factors (TFs) regulating the significantly changed genes (Log2 ratio ≥ 1.0 and FDR ≤ 0.001) were analyzed by YEASTRACT (http://www.yeastract.com/), and the top 20 enriched TFs are summarized in Fig. 3. The enrichment of TFs was almost identical between the two different stages, except that Spt10p only appeared in SCXG vs YBXG, whereas Hsf1p only in SCX vs YBX. Among the top 5 TFs, Ace2p and Sfp1p are involved in regulating cell cycle. Recently, Ace2p and Sfp1p were also identified as the top two enriched transcription factors in a stress-tolerant yeast that was resistant to acetic acid, furfural, and a mixture of the two inhibitors. Moreover, overexpression of *SFP1* or *ACE2* could significantly improve the ethanol productivity or fermentation rate, respectively, under the stress of acetic acid and furfural [25]. However, so far, there is no study on the effect of *ACE2* and *SFP1* on xylose utilization. It will be interesting to further study the regulatory effects of these two transcription factors on xylose metabolism.

Several enriched transcription factors, such as Cst6p, Cin5p, Msn4p and Msn2p, are also related to stress response [26, 27]. Analyzing by YEASTRACT, three of these TFs (Cst6p, Cin5p, Msn2p) are regulators of Yap1p, which is a key TF in oxidative stress tolerance. Yap1p was found to be involved in regulating xylose metabolism regardless of host strain or expressed pathways [4]. In our study, enrichment of these TFs in both fermentation stages implied the presence of a stress response during carbohydrate metabolism of *S. cerevisiae*. *MSN2* and *MSN4* encode general stress response transcription factors which regulate the expression of approximately 200 genes through binding to stress response elements (STREs) in the promoter region. These transcription factors are involved in multiple stress responses such as osmotic shock, oxidative stress, and heat shock, but the expression of *MSN2* is constitutive, whereas *MSN4* is induced by stress [28, 29]. In the present study, no obvious difference in the transcription of *MSN2* was observed during the fermentation of both carbon sources, but *MSN4* was downregulated in YB-2625 when compared to S288C under both stages. As previously reported, a *MSN4* deletion mutant showed improved specific xylose consumption rate by 120% during mixed-sugar fermentation. However, deletion of *MSN2* had no significant effect on xylose metabolism [30]. In addition, Gcn4p was found to be an enriched TF in both stages with the function of activator of amino acid biosynthetic genes. Gcn4p was proposed to play important role in xylose metabolism of the recombinant *S. cerevisiae* strains [4]. Hence, the results here indicate that regulation of the stress response and amino acid metabolism are two important strategies for YB-2625 to response to xylose as a recombinant yeast.

When TFs profiles of both stages were compared, it was found that Spt10p only appeared in SCXG vs YBXG, whereas Hsf1p only in SCX vs YBX. Spt10p is a histone H3 acetylase which is involved in chromatin maintenance and transcriptional regulation and is also required for transcription of some histone genes [31]. Our results suggest that chromatin maintenance in the mixed-sugar utilization stage in *S. cerevisiae* YB-2625 may benefit to keep cell stability. Hsf1p activates hundreds of genes under highly diverse stresses imposed by oxidants, heat, glucose, and diauxic shift. Moreover, it also regulates genes involved in protein folding or refolding and degradation of damaged proteins [32]. During the xylose-utilization stage, cells were in a state of oxidative stress or energy deficiency; therefore, target genes of Hsf1p were significantly changed in YB-2625 to support good cell viability. It will be interesting to further explore the functions of Hsf1p in xylose utilization.

### Differentially transcribed genes involved in carbohydrate metabolism

#### Transporters

Xylose utilization by *S. cerevisiae* is hampered by its inefficient uptake. In yeast cells, there are numerous sugar transporters including Hxt1p to Hxt17p and Gal2p; however, these transporters possess much greater specificity for glucose compared to xylose. Among these genes, a few transporters, including Hxt1p, Hxt2p, Hxt4p, Hxt5p, Hxt7p, and Gal2p, have shown limited ability for xylose transportation [33]. Moreover, xylose uptake is inhibited when glucose is present in the medium, mainly due to the preferential uptake of glucose by transporters. Heterologous xylose transporters are frequently employed to solve the problem, and now, native transporters, such as *HXT7* [34], have been modified to optimize xylose uptake. Hence, we were interested in identifying native transporters with differential expression. Previously, upregulation of sugar transporters (*HXT1*, *HXT2*, *HXT5*, *HXT7, HXT10*, *HXT13*, *HXT15,* and *HXT16*) contributed to elevated *V*_max_ for xylose and reduced the negative impact of glucose on xylose transportation in the evolved yeast strains [35]. For the natural isolate yeast YB-2625, transcription of multiple sugar transporters was relatively more evident when compared to S288C. A moderate glucose transporter *HXT5* was shown to be the most upregulated by 3.64- and 3.72-fold under both carbon source conditions which indicated that it took part in sugar uptake regardless of carbon source. Hxt5p is a special transporter containing a stress-responsive element in the promoter region. Meanwhile, it is the only hexose transporter involved in accumulation or metabolism of reserved carbohydrates, such as trehalose [36]. *HXT5* was induced when utilizing non-fermentable carbon source; therefore, we speculated the higher expression level of the transporter was induced by faster xylose utilization of YB-2625 than S288C, because xylose is recognized as a non-fermentable carbon source for *S. cerevisiae*. We also noticed there were multiple mutation sites in the upstream and CDS region of *HXT5* according to the genome sequence of YB-2625 (Additional file 1: Table S3), which was recently performed by our lab (data not shown). Some of these mutations may be responsible for the variation of transcription. Similarly, Hxt4p and Hxt7p have previously shown the ability to transport xylose [33], and interestingly, both showed enhanced transcription levels during the xylose-utilization stage in this study. During mixed-sugar utilization, the *HXT13* gene was upregulated by 2.2-fold. Hxt13p has shown minor hexose transport activity and is induced by non-fermentable carbon sources, such as glycerol and ethanol [37], but xylose has never been reported to trigger the same effect. *HXT3*, which is induced when cells are grown on either high level or low level of glucose, was also upregulated by 2.89-fold during YBX vs SCX. In brief, the upregulation of *HXT3*, *HXT4*, *HXT5*, *HXT7*, and *HXT13* indicated that the differences between the two strains in sugar uptake might contribute to the difference in glucose and xylose utilization.

#### Differential expression involved in central carbon metabolism

**Fig. 4:**
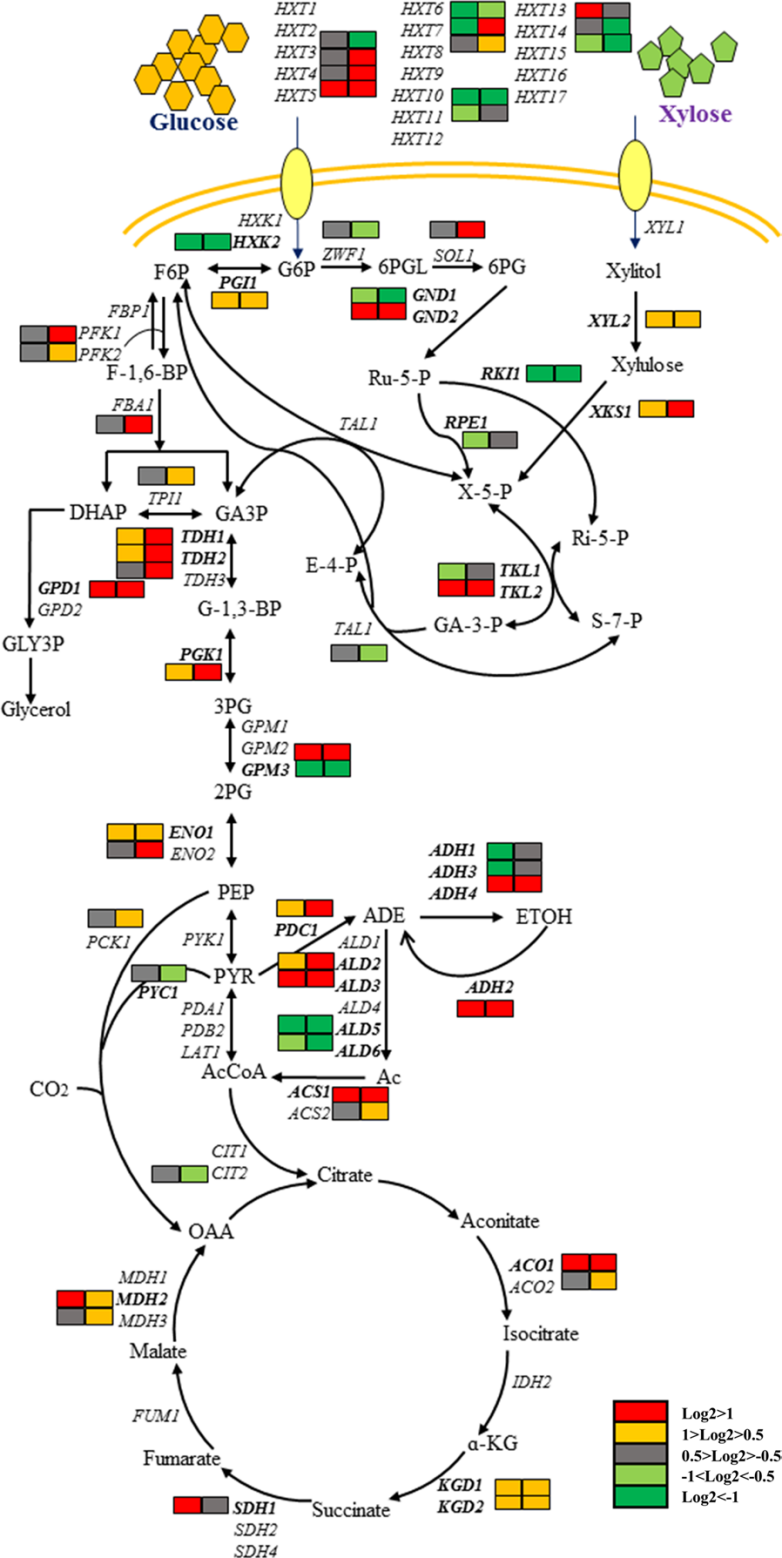
Transcriptional profiling of central metabolic pathways. Varied colors represent for different change levels, and the left and right rectangles stand for YBXG vs SCXG (7 h) and YBX vs SCX (48 h), respectively

A large number of genes related to central carbon metabolism, including glucose consumption and ethanol production, as well as the generation of by-products, showed different transcription levels (Fig. 4). Meanwhile, the endogenous genes involved in xylose utilization were also listed in the network.

In the mixed-sugar fermentation process, transcription levels of glycolysis genes (*PFK1*, *PFK2*, *FBA1*, *TPI1*, *TDH1*, *TDH2*, *TDH3*, *PGK1*, *ENO1,* and *ENO2*) were moderately higher in YB-2625, consistent with more rapid glucose consumption in YB-2625 (Fig. 1a). Enhanced expression of genes involved in glycolysis has been found in an evolved xylose-fermenting recombinant strain in exponential growth phase during mixed-sugar fermentation [38], suggesting a beneficial effect of active glycolysis on xylose utilization. Previously, high rate of carbon flux from fructose-6-phosphate to glycerol-3-phosphate in upper glycolysis pathway has been shown to be beneficial for driving xylose metabolism through the PP pathway [39]. Therefore, the significant upregulation of *FBA1* in the xylose-utilization stage may be conducive to the superior xylose-utilization capability of YB-2625.

As demonstrated in Fig. 4, *PDC1* is vital for ethanol fermentation by encoding the major pyruvate decarboxylase isozyme. Meanwhile, it also takes part in the catabolism of various amino acids [40]. It was reported that dramatically reduced expression of *PDC1* possibly caused low ethanol productivity of a xylose-fermenting recombinant in xylose medium [41]. Therefore, enhanced expression of *PDC1* in YB-2625 compared to S288C may also act as one of the advantages for ethanol production. Upregulation of alcohol dehydrogenase encoding gene *ADH4* was observed during both sugar utilization stages, whereas *ADH2* which catalyzes the reverse reaction converting ethanol to acetaldehyde, was also enhanced by 1.68-fold. Improved ethanol production by 52% was obtained through disruption of *ADH2* in *S. cerevisiae* strain As2.4 [42]. Therefore, we assumed that *ADH2* upregulation may be disadvantageous on xylose utilization of YB-2625. However, deletion of *ADH2* from YB-2625 did not show any effect on xylose consumption (data not shown), suggesting that additional factors are involved in the regulation of xylose consumption.

On the other hand, upregulation of *ALD2*, *ALD3,* and *ACS1* may direct carbon flux to AcCoA and significantly increased expression of genes involved in the TCA cycle (*ACO1*, *KGD1*, *KGD2*, *SHD1*, and *MDH2*) was also observed in both conditions. The TCA cycle acts as one of the targets of transcriptional regulation to optimize xylose utilization [4]. Upregulation of genes involved in the TCA cycle was found in a recombinant xylose-fermenting yeast with deletion of *GRE3* and *PHO13.* This strain also showed better performance of xylose utilization, and the change of these genes (i.e. *GRE3* and *PHO13*) also occurred in evolved strain with higher specific ethanol production rate [43, 44]. According to the comparison between recombinant industrial and laboratory yeast, many of genes encoding TCA cycle enzymes were more inducible in the industrial strain with superior xylose-fermenting ability [17]. Moreover, a 1.25-fold higher specific ethanol production rate could be reached by overexpression of Mdh2p in a xylose-fermenting recombinant *S. cerevisiae* carrying the XR–XDH pathway [45]. *SDH1* was shown to be upregulated in the recombinant yeast strain with a better performance in xylose utilization [4]. Improved expression of *MDH2* and *SDH1* in YB-2625 may contribute to better xylose fermentation. The above-mentioned analyses indicated that an intensive TCA cycle in YB-2625 may be important for xylose metabolism.

#### Gluconeogenesis

From the transcriptomic data, we can see that almost all genes involved in gluconeogenesis (*GPM2*, *TDH2*, *FAB1*, *ENO2*, *PGK1*, *TDH3*, *TDH1*, *MDH2*, *PGI1*, *PCK1*, *TPI1*, *ENO1*, *PYC1*, and *PYC2*) were upregulated during the xylose-utilizing stage (Additional file 1: Table S4). The *S. cerevisiae* expressing XR–XDH pathway required glucose-6-phosphate generated from gluconeogenesis to realize xylose assimilation and enhanced expression of genes related to gluconeogenesis are common for recombinant yeasts in response to xylose [44]. Moreover, other natural yeasts, *Scheffersomyces stipitis* and *Kluyveromyces lactis* also increase the expression of related enzyme activities when utilizing xylose [46]. In other words, the yeasts recycle xylose back to the production of glucose-6-phosphate through the non-oxidative pentose-phosphate pathway to realize xylose metabolism [46]. However, downregulation of key genes in the gluconeogenesis pathway was also revealed in an evolved yeast with improved xylose-utilization ability [11], suggesting that differential strategies were adopted by the natural strain and lab-evolved yeast. Taken together, higher flux rate of gluconeogenesis is one of the distinguishing features of YB-2625 from the laboratory strain S288C.

#### Endogenous xylose consumption pathway

Until now, no native *S. cerevisiae* strain, even no genetically engineered strains could metabolize xylose as efficient as glucose. Many studies have focused on introducing a heterologous xylose metabolic pathway into the native *S. cerevisiae* strains, mainly including the xylose reductase/xylitol dehydrogenase (XR–XDH) pathway and xylose isomerase (XI) pathway [47]. Endogenous xylose-assimilating genes are also revealed to contribute to superior xylose utilization [48]. There are multiple aldo–keto reductases which are thought to have the function of converting xylose to xylitol, including *GRE3*, *GCY1*, *YPR1*, *YJR096W*, and *YDL124W*, whereas *XYL2*, *SOR1,* and *SOR2* were found to have similar function with xylitol dehydrogenase to convert xylitol to xylulose [14]. An endogenous xylose utilization gene cassette composed of aldose reductase (*GRE3*), sorbitol dehydrogenase (*SOR1*), and xylulose kinase (*XKS1*) has been successfully employed to construct *S. cerevisiae* strains with efficient xylose-fermenting ability [48]. YB-2625 showed higher expression of genes that may enable xylose reduction; *GRE3*, *GCY1,* and *YPR1* were induced 2.16-, 0.71-, and 0.73-fold compared to S288C at 7 h, while *YPR1* was upregulated 0.75-fold at 48 h (xylose-utilization stage). The enhanced expression of *XYL2* by roughly 0.5-fold was revealed in YB-2625 compared to S288C in both investigated stages. It is interesting that the natural yeast isolated from bagasse has enhanced expression of endogenous genes for xylose metabolism. This trait may have evolved to help it survive in that environment.

Intriguingly, the FPKM values of *SOR1* and *SOR2* were extremely low in both strains (Additional file 1: Table S5). Therefore, it is reasonable to suppose that endogenous dehydrogenase is a bottleneck for natural or wild yeasts to metabolise xylose. On the other hand, a higher expression level of *XKS1* by 0.93- and 1.69-fold, respectively, in YB-2625 in the two investigated stages was observed. The activity of xylulose kinase was considered as the main bottleneck for xylose-fermenting strains [49]; therefore, increased transcription of *XKS1* could be regarded as a characteristic of suitable strains for xylose fermentation. Endogenous *XKS1* was more highly expressed in industrial yeast than in laboratory strains with weaker xylose or glucose fermentation performance [14]. Hence, we assume that xylulose kinase, as well as endogenous enzymes bearing xylose reductase activities, are the key players for xylose consumption in YB-2625.

It is worth noting that the transcription level of *TKL2* was roughly threefold higher in YB-2625 in the two stages. *TKL2* is located on the non-oxidative branch of the pentose-phosphate pathway to encode transketolase, but it is a minor isoform [50, 51]. It will be interesting to explore the effect of *TKL2* overexpression on xylose utilization in different yeast hosts.

The major character of the natural xylose-utilizing yeasts is the production of xylitol, and accumulation of xylitol is also one of the major problems for engineered *S. cerevisiae* in the process of xylose fermentation. *FPS1* is an aquaglyceroporin involved in the export of xylitol in *S. cerevisiae*, and 71% decreased xylitol yield, and nearly four times of ethanol yield were obtained when deleting *FPS1* in a yeast strain with unbalanced XR and XDH activities [52]. Downregulation of *FPS1* by − 0.86-fold in YB-2625 in the condition of YBX vs SCX was found. Another key gene, *PHO13*, was downregulated by 3.09-fold in YB-2625 strain under the condition of YBXG vs SCXG. *PHO13* encodes alkaline phosphatase specific for p-nitrophenyl phosphate. Recently, it was hypothesized that dephosphorylation of Sedoheptulose-7P by *PHO13* might cause carbon loss and inefficient xylose utilization [53]. The deletion of *PHO13* can be employed in constructing xylose-fermenting *S. cerevisiae* for enhanced ethanol production. In summary, the lower expression level of *FPS1* and *PHO13* may contribute to superior xylose utilization in YB-2625.

Except for xylose, YB-2625 also presented catabolism ability of glycerol (Fig. 1), which is another non-fermentable carbon source to *S. cerevisiae*. Consistent with the glycerol consumption capability of YB-2625, genes involved in the major pathway of glycerol catabolism were upregulated in YB-2625. *STL1*, which encodes a glycerol/H^+^ symporter, showed 1.85-fold upregulation in the xylose-utilization stage. *GUT1* and *GUT2* which encode glycerol kinase and glycerol 3-phosphate dehydrogenase, respectively, also showed enhanced transcription (0.86-, 0.42-fold at 7 h, and 0.73-, 1.44-fold at 48 h, respectively). The results here indicate a high diversity between natural and laboratory yeast in carbohydrate metabolism.

#### Varied expression of carbohydrate metabolism-related transcription factors

**Fig. 5:**
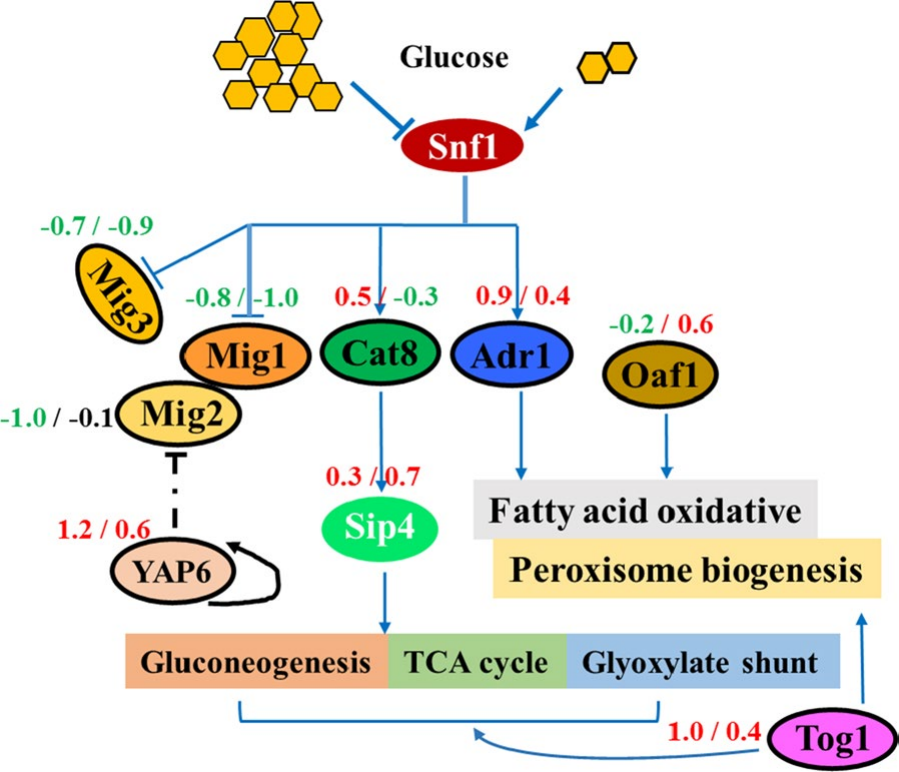
Network of selected transcription factors related to carbon metabolism. The TFs with obvious change were labeled by corresponding variations, and figures in the left and right represent the condition of YBXG vs SCXG (7 h) and YBX vs SCX (48 h), respectively

Transcription factors are essential for cells to regulate gene expression. Multiple gene expression variations occurred during the diauxic shift. The shift is regulated by various transcription factors to realize metabolism of the non-fermentative carbon sources after glucose is exhausted [54, 55]. The general network of transcription factors was demonstrated in Fig. 5, Snf1 kinase plays the central role in non-fermentable carbon source utilization, and it could target the related transcription factors. Mig1p and Mig3p are inactivated in response to glucose depletion, while Mig2p co-operated with Mig1 in the process. In accordance with the shift of carbon sources, when comparing xylose fermentation with mixed-sugar fermentation stage, *MIG1*, *MIG2,* and *MIG3* were all downregulated in both yeast strains. It is interesting to note that lower transcription levels of all the three *MIG* genes were shown in YB-2625, which were − 0.77-, − 1.04-, and − 0.70-fold decreased in the case of YBXG vs SCXG. Even under the condition of xylose-utilization stage (YBX vs SCX), the *MIG1* and *MIG3* genes were also downregulated (− 1.03, − 0.87). In addition, *HXK2* in *S. cerevisiae* is not only a predominant glucose kinase but also a regulator of gene transcription. It was found that *HXK2* was downregulated by 2.84-fold under the condition of YBXG vs SCXG. Hxk2p acts as an intracellular glucose sensor under high glucose concentration which is required for inhibiting Mig1p phosphorylation to stabilize the repressor complex [56]. Downregulation of *HXK2* may benefit relief of glucose expression in YB-2625.

Although we only quantified transcription levels in this study, the weaker expression of glucose depression-related genes in YB-2625 discussed above compared with that in S288C suggested that this wild yeast was more robust to overcome the glucose repression effect. Simultaneous co-fermentation of mixed sugar present in lignocellulosic hydrolysates is a promising strategy for cellulosic ethanol production, because lower yield and productivity of ethanol is obtained when strains prefer utilization of glucose to other sugars [57]. Many strategies to increase co-consumption of mixed sugars have been attempted. For instance, *HXK2* deletion caused co-consumption of galactose and sucrose with glucose, while *MIG1* deletion led to the simultaneous utilization of glucose and sucrose [57, 58]. In the case of xylose–glucose co-fermentation, a *MIG1* deletion strain showed higher xylose consumption compared to the control before glucose depletion in the progress of batch cultivation. Moreover, a 25% increased xylose consumption rate accompanied by an 11% increased ethanol yield by *MIG1* deletion was obtained during continuous cultivations in mixed sugars [59]. Unfortunately, although mixed sugar could be co-consumed through *MIG1* and *HXK2* deletion, there were some negative effects such as reduced glucose uptake rate and prolonged log phase [57]. We assumed that the expression of both genes was lower in YB-2625 than S288C, but this lower expression did not affect the growth of the natural isolate. As suggested by the study previously, high expression level of *HXK2* was not required for efficient xylose fermentation [11]. Therefore, the lower expression of *HXK2* may act as one of the factors for weakened glucose repression and improved xylose-utilization ability of YB-2625. Recently, it was reported that 60% improvement of specific xylose consumption rate was found in *HXK2* deletion yeast strain when performing mixed-sugar fermentation. The decrease of HXK activity could increase flux through the xylose consumption pathway (XDH, XK), glyoxylate shut, and TCA cycle, which was beneficial for xylose consumption [60]. In addition, higher expression levels of transporters including *HXT2*, *HXT4*, and *HXT7*, which are functional in xylose transport, was previously identified in *HXK2*-deficient *S. cerevisiae* [61]. Therefore, the naturally decreased expression of *HXK2* is also one of the positive characteristic of YB-2625 for superior xylose consumption ability.

On the other hand, *MIG2* showed the most dramatic changes among the three genes; therefore, the TFs that putatively regulate its expression were further analyzed using YEASTRACT. As shown in Additional file 1: Figure S2, Yap6p was the most obviously upregulated TF under the condition of YBXG vs SCXG. However, there are still no experimental data on the regulation of *MIG2* by Yap6p. Here, we speculated that the expression of *MIG2* was inhibited by Yap6p for the reason that upregulation of *YAP6* by 1.24-fold was revealed together with the downregulation of *MIG2* by 1.04-fold. Yap6p is a TF associated with salt tolerance and predicted to have a role in the regulation of expression of genes involved in carbohydrate metabolism [62]. It will be interesting to further explore the function of *YAP6* in the process of xylose metabolism.

Carbohydrate metabolism is regulated by various TFs in *S. cerevisiae*. For instance, deletion of respiratory regulatory gene *CAT8* was beneficial to improve specific glucose consumption rate and decrease the generation of the by-product acetic acid, and then increase the specific rate of ethanol production by 22% [30]. However, the influence of *CAT8* deletion on xylose metabolism has still not been identified in *S. cerevisiae*. Mutation of another respiratory regulatory gene *HAP4* conferred improved specific xylose consumption rate by 170% in *S. cerevisiae* [30]. In our study, no clear variation was found in these two TF encoding genes on their transcription level, and it will be interesting to examine the specific regulation mode of different yeast strains. In addition, Tog1p is another transcriptional activator moderating genes for several other aspects mainly about β-oxidation of fatty acid, glyoxylate shunt, and gluconeogenesis (Fig. 5), such as *POX1*, *FOX2*, *POT1*, *IDP2*, *MLS1*, *ICL1*, *FBP1,* and *PCK1*, to regulate the utilization of non-fermentable carbon sources such as ethanol, glycerol, or acid [63]. Therefore, the higher expression of *TOG1* by 1.02- and 0.35-fold in the two stages, respectively, as well as the upregulation of nearly all of its regulated genes (Additional file 1: Figure S3) during both stages might have contributed to specific mechanisms of carbohydrate metabolism of the natural yeast YB-2625.

### Stress response-related changes in the transcriptomic data

#### Antioxidant enzyme-related genes and ROS determination

Xylose toxicity has been observed even in engineered strains when cultivated in the presence of xylose at a concentration higher than 10 g/L [8]. Increased expression of oxidative stress-responsive genes was shown in the recombinant *S. cerevisiae* [14]. Similarly, ROS and damaged cellular constituents accumulated when yeast was cultured in the media containing fructose [64]. However, the cytotoxic impact of xylose on yeast cultivation remains unclear.

**Fig. 6:**
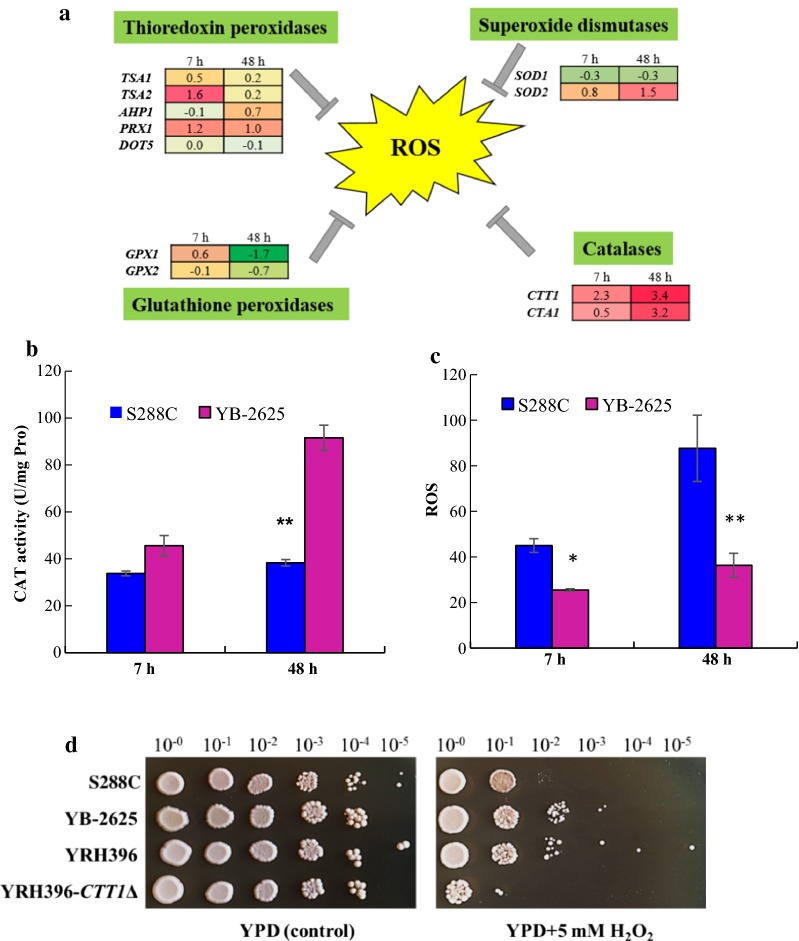
Relative transcription levels of genes encoding ROS detoxifiers between *S. cerevisiae* YB-2625 and *S. cerevisiae* S288C in 7 and 48 h. **a** Different expression of ROS detoxifiers including thioredoxin and glutathione peroxidases, superoxide dismutases, as well as catalase. Red and green rectangle stand for the condition of upregulation and downregulation, respectively; **b** catalase activity in *S. cerevisiae* YB-2625 and *S. cerevisiae* S288C. *p < 0.05; **p < 0.01. **c** ROS level in *S. cerevisiae* YB-2625 and *S. cerevisiae* S288C in 7 h and 48 h as the samples of transcriptomic analysis; **d** cell growth ability of the wild-type yeast strains, xylose-fermenting recombinant strain YRH396, and the *CTT1*-deleting mutant of YRH396 under the stress of 5 mM H_2_O_2_, and YPD agar medium without H_2_O_2_ addition was used as a control

In this study, genes involved in the oxidative stress response and detoxification of ROS were analyzed (Fig. 6a). Antioxidant enzymes present in yeast cells include superoxide dismutases, catalases, glutathione peroxidases, and thioredoxin peroxidases [64]. Superoxide dismutases encoded by *SOD1* and *SOD2* play important roles in oxygen radical detoxification. After that, the generated hydrogen peroxide could be catalyzed by catalase A located at cytosol and peroxisomes, respectively, which are encoded by *CTT1* and *CTA1*. Both catalases can break down hydrogen peroxide into dioxygen and water molecules, protecting yeast cells from oxidative damage [65, 66]. The correlation between these ROS-scavenging enzymes and oxidative stress resistance was summarized previously [67]. Differences in the transcription of genes in antioxidant system between the two strains were compared and presented in Fig. 6a. Notably, the most significant diversity was shown in *CTT1* and *CTA1*; specifically, *CTT1* was with enhanced expression of 2.3- and 3.4-fold during both stages, respectively. Meanwhile, *CTA1* was elevated by 3.2-fold in the condition of YBX vs SCX. The expression of *CTA1* and *CTT1* could be induced by peroxide stress and increased catalase activity was obtained by pretreatment with low concentration of H_2_O_2_ [68].

Yeast cells contain several peroxidases which are capable of reducing hydrogen peroxide formed by superoxide dismutase. Thioredoxin peroxidases encoded by *TSA1* and *TSA2* are cytoplasmic location, and enzyme encoded by *DOT5* is nuclear location. Prx1p is the 1-Cys peroxiredoxin and functional as a peroxidase, and is located in the mitochondria. In this study, *PRX1* was upregulated by 1.2- and 1.0-fold in both sugar utilization stages. Specific increase in expression for only the mitochondrial peroxiredoxin may indicate that redox stress induced by xylose is highest in that organelle. Consistent with this observation, *SOD2*, a highly conserved gene encoding a manganese-superoxide dismutase located in the mitochondrial matrix, was also enhanced by 0.8- and 1.5-folds under the two conditions. High cell sensitivity to ethanol and heat shock could be caused by lacking of *SOD2* [69]. The results here indicate that the wild-strain YB-2625 has stronger expression of genes related to ROS-scavenging capability than laboratory strain S288C to protect yeast cells via superoxide dismutases and catalase.

We further detected antioxidant enzyme activities of the two strains in the conditions of YBXG vs SCXG and YBX vs SCX. In the xylose-utilization stage, the CAT activity of YB-2625 was 1.9 times higher than that of S288C (Fig. 6b), whereas no significant difference was observed between YB-2625 and S288C in CAT activity in the condition of mixed-sugar stage. However, no obvious change was found in SOD activity between the two strains and both were at a low level (data not shown). We assumed that higher catalase activity in YB-2625 v.s. S288C led to lower ROS level. Connection between catalase activity and oxidative tolerance was confirmed by spot assay results (Fig. 6d). It was confirmed that the native oxidative tolerance of YB-2625 was higher than that of S288C. Recently, the cytosolic catalase, Ctt1p, was shown to provide the majority of catalase activity in the cell [70]. To confirm that *CTT1* plays an important role in oxidative tolerance of *S. cerevisiae* YB-2625 derived recombinant strain YRH396, a *CTT1* gene deletion strain was evaluated. When *CTT1* was deleted, H_2_O_2_ tolerance of the strain was decreased sharply, indicating that *CTT1* was also the dominant form of catalase in the YB-2625 genetic background. After that, intracellular ROS level of the two strains was determined. As shown in Fig. 6c, the ROS level of YB-2625 was significantly lower than the control S288C in both stages investigated in this study. In the condition of YBXG vs SCXG, the ROS level of YB-2625 was 43.3% lower than S288C, while in the next condition, it was 58.6% lower than the control. Meanwhile, it was also observed that YB-2625 kept steady ROS level, but in S288C, the ROS level was increased along with the fermentation process, which indicated that higher ROS could be caused by xylose fermentation. The genetic background of this wild strain is distinctive from the lab yeast in the aspect of ROS-scavenging ability, possibly relating to the environment where this strain was separated. Based on the results obtained in the study, it is reasonable to hypothesize that the enhanced stress tolerance and ROS-scavenging ability of YB-2625 strain is one of the important factors to promote xylose utilization.

**Fig. 7:**
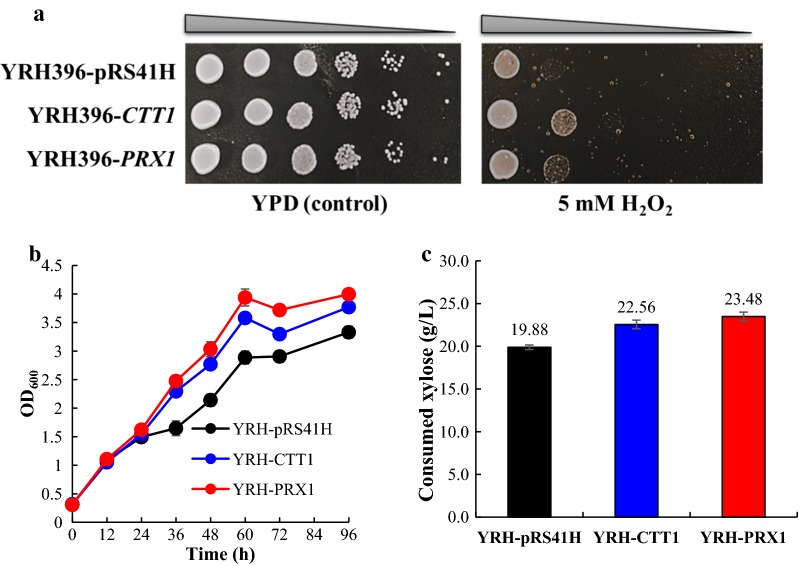
Effects of *CTT1* and *PRX1* overexpression on xylose-fermenting ability of the recombinant strain YRH396. **a** Cell growth ability of the yeast strains with *CTT1* or *PRX1* overexpression, and the transformants with the empty vector pRS41H served as a control. YPD agar medium supplemented with 5 mM H_2_O_2_ was applied to test the oxidative stress tolerance ability of the different strains, and YPD agar medium was used as a control. **b** Growth ability of the *CTT1* or *PRX1* overexpression strains compared to the control strain with the control empty vector. Ethanol fermentation was performed with 4 g/L yeast extract, 3 g/L peptone, and 40 g/L xylose. **c** Xylose consumption of the yeast strains after fermentation for 96 h. The results shown were the mean values of triplicate experiments

Subsequently, we performed *CTT1* and *PRX1* overexpression in a xylose-fermenting recombinant YRH396 to test whether the above-mentioned hypothesis is reasonable. Oxidative tolerance ability of YRH396-pRS41H (control), YRH396-*CTT1,* and YRH396-*PRX1* was compared under the stress of 5 mM H_2_O_2_ (Fig. 7a). Improvement of growth of the overexpressed strains was observed, indicating the enhanced *CTT1* and *PRX1* expression benefits ROS-scavenging ability. To investigate the effect of enhanced oxidative tolerance on xylose fermentation ability, the strains were further examined using 40 g/L xylose as the sole carbon source. Both mutants exhibited increased growth compared to the control in the fermentation process. The final OD_600_ of the two strains, YRH396-*CTT1* and YRH396-*PRX1*, was improved to 3.77 and 4.00 compared to the control value of 3.33 (Fig. 7b). What is more, the two mutants consumed 22.56 and 23.48 g/L xylose, respectively, after fermenting for 96 h, 13.5 and 18.1%, respectively, higher than that consumed by the control (19.88 g/L) under the same culture conditions (Fig. 7c). These results demonstrated that enhanced expression of *CTT1* and *PRX1* could improve xylose consumption, indicating a positive correlation between oxidative stress tolerance and xylose-utilization ability. However, when *CTT1* and *PRX1* overexpression was tested using the recombinant strain with the *S. cerevisiae* BY4741 background, no effect on xylose utilization was observed despite improved growth (data not shown). These results indicate that improved xylose metabolism by *CTT1* and/or *PRX1* overexpression is host-dependent, which is also reported in the previous study [4]. It was suggested that decreased transcription level of *SOD1* in the INV host led to poorer xylose utilization comparing to the CTY host [4]. We thus deduce that different host strains may employ different mechanisms against oxidative stress. Other endogenous factors which also contribute to improved xylose utilization were also reported, such as *ASK10* [9], *CYC8* [35], *ISU1* [71], *SSK2* [71], as well as epistatic interactions of *IRA2* and *ISU1* [72]. We assume that different endogenous genetic elements may function synergistically to adapt to xylose metabolism. Further studies on regulation of xylose consumption by endogenous genes will provide more insights on rational development of xylose-utilizing yeast.

Xylitol is always generated during xylose fermentation in wild-type *S. cerevisiae*, and hence, we predicted that xylitol is one of the factors inducing oxidative stress response. When *S. cerevisiae* YB-2625 was exposed to xylitol treatment (10 g/L xylitol) for 3 h, the transcription levels of *CTT1* and *CTA1* were upregulated when compared to the non-treatment control (Additional file 1: Figure S4), indicating the increased detoxification of ROS. Interestingly, *HSP12* was shown to be downregulated by xylitol treatment. *HSP12* is a gene responsible for DNA replication which could maintain membrane organization during stress conditions [73]. Higher expression of *CTT1*, *CTA1* along with lower transcription level of *HSP12,* indicated that xylitol may cause oxidative stress but not DNA replication stress. We also analyzed genes involved in membrane components and discussed in the below text.

#### Genes involved in ergosterol metabolism

Sterol is an important membrane component to keep cell structure by moderating fluidity and stability. In fact, many transporters are located in membrane lipid to affect transportation of multiple substances, while toxic substances could influence the integrity of plasma membrane. In addition, sterol is crucial for other cell functions such as chaperone, protein modification, second messenger, and signal receptor [74, 75]. Sterols are important for many bioprocesses, playing a vital role in adaptation to various environments [76]. Ergosterol is a predominant sterol in yeast [77]. ROS content in *S. cerevisiae* was decreased when supplementing ergosterol in the medium, and then, increased cell viability and superoxide activity were obtained accompanied by a reduction of oxidative damage to membranes and proteins [78]. Ergosterol is also beneficial to improve resistant ability towards ethanol toxicity, maintaining an optimal membrane thickness during anaerobic fermentation. Furthermore, supplementation of ergosterol together with Tween 80 could dramatically improve growth rate and xylose consumption rate by 70 and 50%, respectively [18, 77], suggesting the importance of ergosterol in xylose utilization.

**Fig. 8:**
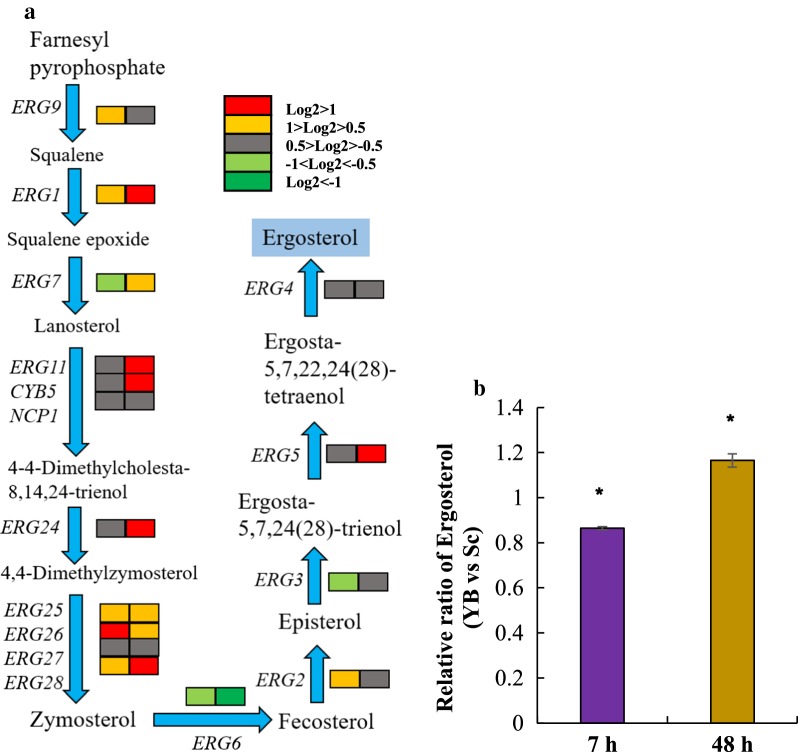
Differences of ergosterol biosynthesis between the two strains. **a** Genes involved in ergosterol biosynthesis, and rectangles in the left and right position stand for 7 h and 48 h, respectively; **b** Relative ratio of ergosterol between *S. cerevisiae* YB-2625 and *S. cerevisiae* S288C in the different stages of transcriptomic analysis. *p < 0.05

In our study, expression of ergosterol biosynthesis genes including *ERG1*, *ERG11*, *CYB5*, *ERG24*, *ERG25*, *ERG26*, *ERG28,* and *ERG5*, was all enhanced in YB-2625 compared with S288C in the condition of YBX vs SCX. However, in the condition of YBXG vs SCXG, most of the genes involved in ergosterol biosynthesis were just slightly upregulated and some of the genes were downregulated, such as *ERG7*, *ERG6,* and *ERG3*, as shown in Fig. 8a. These upregulated genes were important for ergosterol biosynthesis; for instance, *ERG1* encodes squalene epoxidase, which plays an essential role in the pathway. Subsequently, ergosterol contents of YB-2625 and S288C were detected using cells in 7 h and 48 h, respectively. Consistent with the changes in transcription levels, we, indeed, detected increased content of ergosterol in YB-2625 by 20% compared with that of S288C in 48 h (Fig. 8b). Therefore, we hypothesized that higher expression level of genes related to ergosterol biosynthesis of YB-2625 in the stage of xylose fermentation contributed to the decreased ROS level as well as superior xylose utilization.

## Conclusions

**Fig. 9:**
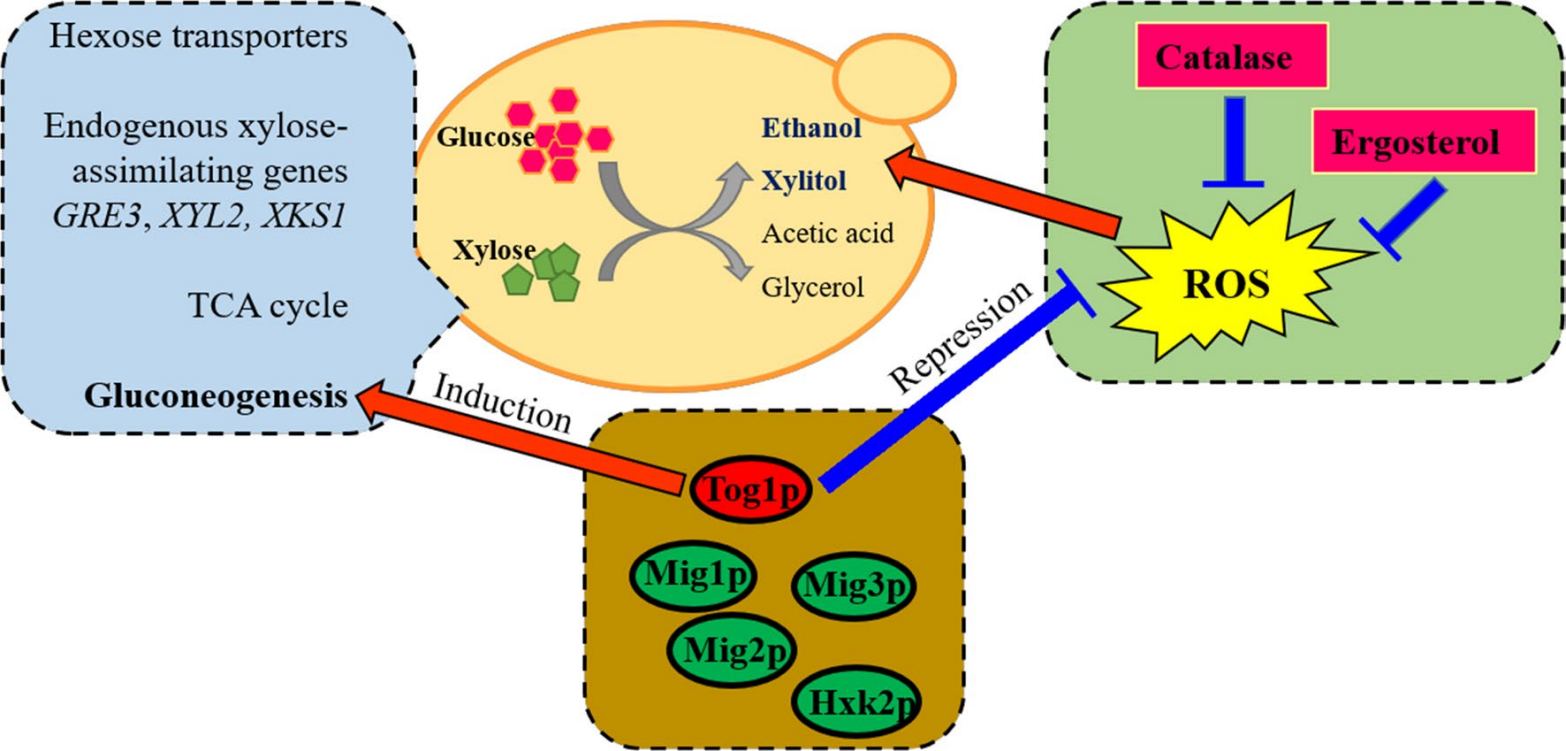
Proposed model for superior performance of *S. cerevisiae* YB-2625 in the aspects of xylose fermentation and stress response. Three modules were highlighted in different colors. Module 1 includes transporters, endogenous xylose-assimilating genes, and metabolic pathway (in blue) which were well studied. Module 2 emphasizes the key transcription factors (in brown) and module 3 contains key factors involved in oxidative stress tolerance (green)

Comparative transcriptomic analysis between the natural yeast YB-2625 isolated from bagasse and the model yeast S288C was conducted in the process of mixed-sugar fermentation with glucose and xylose. It was found that besides enhanced expression of genes involved in glycolysis, TCA cycle, as well as gluconeogenesis, the wild strain YB-2625 also showed higher expression of hexose transporters and endogenous xylose metabolism related genes, such as *GRE3*, *XYL2,* and *XKS1* to realize more efficient xylose metabolism than the laboratory strain. Moreover, weakened expression of glucose repression related transcription factors *MIG1*, *MIG2*, *MIG3*, and *HXK2* may also contribute to the accelerated xylose utilization. Sustained low ROS and upregulation of oxidative stress resistance genes, such as *CTT1*, *CTA1*, *SOD2,* and *PRX1,* were present in this superior xylose-utilizing yeast. The combination of lower ROS levels, higher catalase activity, and ergosterol content, suggests that improved oxidative stress tolerance was beneficial for xylose utilization of the yeast strain. We further confirmed that *CTT1* and *PRX1* overexpression improved xylose utilization. The proposed mechanisms for superior xylose utilization are presented in Fig. 9. The study here provides new insights into the effects of host genetic background on xylose utilization. Our results also benefit further development of xylose-utilizing recombinant yeast strains for efficient lignocellulosic biofuels and biochemical production.

## Additional file

**Additional file 1: Table S1.** Primers used in this study. **Table S2.** Comparison of Log2 fold changes of selected genes between RNA-seq and qPCR analyses. **Table S3.** Upstream and missense gene variants of *HXT5*. **Table S4.** Change fold of genes involved in gluconeogenesis in the two stages between *S. cerevisiae* YB-2625 and *S. cerevisiae* S288C. **Table S5.** Changed genes shown in the main text. **Figure** S1. Fermentation performance of *S. cerevisiae* YB-2625 and *S. cerevisiae* S288C during mixed-sugar fermentation and overview of the transcriptomic data. A), Growth ability of the two strains; B) and C), comparison of xylitol and ethanol production of the two strains; D), Number of different expression genes under the conditions of YBXG vs SCXG and YBX vs SCX. **Figure S2.** Transcriptional regulatory network between Mig2p and other transcription factors. **Figure S3.** Heat map of the genes regulated by *TOG1* revealed in the comparative transcriptomic analysis data. **Figure S4.** Effect of xylitol treatment on transcription of key genes in *S. cerevisiae* YB-2625.

## Abbreviations

ROS: reactive oxygen species
RNA-seq: RNA sequencing
TCA: tricarboxylic acid cycle
RT-qPCR: real-time quantitative polymerase chain reaction
CAT: catalase
HPLC: high-performance liquid chromatography
GO: Gene Ontology
AcCoA: acetylcoenzyme A
PPP: pentose-phosphate pathway
HSP: heat shock protein
TF: transcription factor
SGD: Saccharomyces Genome Database
YEASTRACT: Yeast Search for Transcriptional Regulators And Consensus Tracking
MIPS: The Munich Information Center for Protein Sequences
