# Supplementary material for: Association of improved oxidative stress tolerance and alleviation of glucose repression with superior xylose-utilization capability by a natural isolate of Saccharomyces cerevisiae
Source: Biotechnol Biofuels. 2018 Feb 5;11:28. doi: 10.1186/s13068-018-1018-y (PMC5798184; doi:10.1186/s13068-018-1018-y)
Supplement: Supplementary file 1 — Additional file 1: Table S1. Primers used in this study. Table S2. Comparison of Log2 fold changes of selected genes between RNA-seq and qPCR analyses. Table S3. Upstream and missense gene variants of HXT5. Table S4. Change fold of genes involved in gluconeogenesis in the two stages between S. cerevisiae YB-2625 and S. cerevisiae S288C. Table S5. Changed genes shown in the main text. Figure S1. Fermentation performance of S. cerevisiae YB-2625 and S. cerevisiae S288C during mixed-sugar fermentation and overview of the transcriptomic data. A), Growth ability of the two strains; B) and C), comparison of xylitol and ethanol production of the two strains; D), Number of different expression genes under the conditions of YBXG vs SCXG and YBX vs SCX. Figure S2. Transcriptional regulatory network between Mig2p and other transcription factors. Figure S3. Heat map of the genes regulated by TOG1 revealed in the comparative transcriptomic analysis data. Figure S4. Effect of xylitol treatment on transcription of key genes in S. cerevisiae YB-2625. [file 13068_2018_1018_MOESM1_ESM.docx]

Supplementary data for

**Association of improved oxidative stress tolerance and alleviation of glucose repression with superior xylose utilization capability by a natural isolate of *Saccharomyces cerevisiae***

Cheng Cheng^2^, Ruiqi Tang^1^, Liang Xiong^2^, Ronald E. Hector^3^, Fengwu Bai^1^, Xinqing Zhao^1*^

^1^State Key Laboratory of Microbial Metabolism, School of Life Sciences and Biotechnology, Shanghai Jiao Tong University, Shanghai 200240, China.

^2^School of Life Science and Biotechnology, Dalian University of Technology, Dalian 116024, China.

^3^Bioenergy Research Unit, National Center for Agricultural Utilization Research, USDA-ARS, Peoria, Illinois, USA.

**Correspondence:** Prof. Xinqing Zhao, **E-mail**: [xqzhao@sjtu.edu.cn](mailto:xqzhao@sjtu.edu.cn). Tel: +86-21-34206673, Fax: +86-21-34208028.

**Table S1** Primers used in this study for RT-qPCR analysis.

| **Primer name** | **Sequence (5’-3’)** |
| --- | --- |
| *ACT1-*F | GCCGAAAGAATGCAAAAGGA |
| *ACT1*-R | GGAAGGTAGTCAAAGAAGCCAAGA |
| *CTT1-*F | CAATTGCCCGTCAACAGA |
| *CTT1-*R | ATTTGGCTCAGGACCGAA |
| *HSP12-*F | CTCTCAAAAGTCATACGCTGAACAA |
| *HSP12-*R | CGTGGACACCTTGGAAGACA |
| *XKS1-*F | CACCCCTCTCCGAACTATCA |
| *XKS1-*R | CATCGTGCCTCTTGTCTTTG |
| *ADH4-*F | TACTAACGGTGGGGAAATCG |
| *ADH4-*R | AGAGGCGGTGGAAACATAAG |
| *HUG1-*F | TCAATGAGCAACCGTGTCAA |
| *HUG1-*R | CAATGATGTTGGCAGAAGGAAC |
| *PRX1-*F’ | CCAAGCTTATGTTTAGTAGAATTTGTAGC |
| *PRX1-*R’ | CCTTAATTAATTATTTCGACTTGGTGAATC |
| *CTT1-*F’ | CCAAGCTTATGAACGTGTTCGGTAAAAAAG |
| *CTT1-*R’ | CCTTAATTAATTAATTGGCACTTGCAATGG |
| *hph*-in-R’ | TTGGTCAAGACCAATGCGGA |

**Table S2** Comparison of Log2 fold changes of selected genes between RNA-seq and qPCR analyses

|  | **RNA-seq** | | **RT-qPCR** | |
| --- | --- | --- | --- | --- |
|  | **Mixed sugars** | **xylose** | **Mixed sugars** | **xylose** |
| ***CTT1*** | 11.39 | 2.61 | 4.96 | 10.85 |
| ***HSP12*** | 6.77 | 5.43 | 3.97 | 5.43 |
| ***XKS1*** | 11.24 | 1.61 | 1.91 | 1.69 |
| ***ADH4*** | 1.27 | 2.95 | 3.03 | 2.50 |
| ***HUG1*** | 0.44 | 0.04 | 0.00 | 0.32 |

**Table S3** Upstream and missense gene variants of *HXT5*.

| Mutate type | Reference base | YB-2625 base | Codon mutate | aa mutate | Position  ChrVIII |
| --- | --- | --- | --- | --- | --- |
| Upstream gene variant | G | A |  |  | 298,776 |
| Upstream gene variant | C | T |  |  | 298,784 |
| Upstream gene variant | T | A |  |  | 298,950 |
| Upstream gene variant | C | G |  |  | 299,066 |
| Synonymous | A | G | AAA<->AAG | K<->K | 294,768 |
| Synonymous | C | T | GGC<->GGT | G<->G | 294,900 |
| Synonymous | C | T | ATC<->ATT | I<->I | 294,915 |
| Synonymous | C | T | GCC<->GCT | A<->A | 295,041 |
| Synonymous | A | C | GTA<->GTC | V<->V | 295,200 |
| Synonymous | C | T | CTG<->TTG | L<->L | 295,376 |
| Synonymous | T | C | TTG<->CTG | L<->L | 295,445 |
| Synonymous | G | A | TTG<->TTA | L<->L | 295,470 |
| Synonymous | C | T | TTC<->TTT | F<->F | 295,620 |
| Nonsynonymous | A | C | AAA<->AAC | K<->N | 296,229 |

**Table S4** Change fold of genes involved in gluconeogenesis in the two stages between *S. cerevisiae* YB-2625 and *S. cerevisiae* S288C.

| **Gene** | **7 h** | **48 h** |
| --- | --- | --- |
| *GPM2* | 2.99 | 6.28 |
| *TDH2* | 1.42 | 4.69 |
| *FBA1* | 0.86 | 2.66 |
| *ENO2* | 0.81 | 2.46 |
| *PGK1* | 1.42 | 2.20 |
| *TDH3* | 1.15 | 2.20 |
| *TDH1* | 1.68 | 2.00 |
| *MDH2* | 2.58 | 1.96 |
| *PGI1* | 0.96 | 1.65 |
| *PCK1* | 1.00 | 1.60 |
| *TPI1* | 1.16 | 1.57 |
| *ENO1* | 1.72 | 1.57 |

**Table S5** Changed genes shown in the main text.

| **Gene** | **Sc-XG-F** | **YB-XG-F** | **Log2** | **P-value** | **Sc-X-F** | **YB-X-F** | **Log2** | **P-value** | **Functional category** |
| --- | --- | --- | --- | --- | --- | --- | --- | --- | --- |
| *MIG1* | 395.70 | 231.27 | -0.77 | 9.05E-83 | 172.40 | 84.21 | **-1.03** | 1.62E-59 | Transcription factor |
| *MIG2* | 185.47 | 90.36 | -1.04 | 2.44E-48 | 54.55 | 51.69 | **-0.08** | 0.52 |  |
| *MIG3* | 91.82 | 56.41 | -0.70 | 6.60E-14 | 14.53 | **7.97** | **-0.87** | 0.00037 |  |
| *HXK2* | 166.66 | 23.28 | -2.84 | 1.4E-219 | 146.46 | 28.12 | **-2.38** | 1.3E-160 |  |
| *YAP6* | 12.29 | 29.05 | 1.24 | 2.21E-11 | 27.12 | 40.60 | **0.58** | 2.35E-05 |  |
| *ADR1* | 45.41 | 84.94 | 0.90 | 1.28E-62 | 120.18 | 153.95 | **0.36** | 2.86E-23 |  |
| *CAT8* | 57.83 | 80.81 | 0.48 | 1.96E-22 | 51.30 | 41.75 | **-0.30** | 9.07E-07 |  |
| *TOG1* | 20.00 | 40.48 | 1.02 | 9.94E-23 | 36.12 | 46.11 | **0.35** | 0.000034 |  |
| *RPI1* | 48.76 | 14.36 | -1.76 | 3.84E-31 | 66.25 | 130.71 | **0.98** | 1.4E-34 |  |
| *MSN2* | 15.66 | 20.06 | 0.36 | 0.01 | 43.68 | 36.04 | -0.28 | 0.003 |  |
| *MSN4* | 489.66 | 183.38 | -1.42 | 0 | 496.03 | 358.96 | -0.47 | 4.30E-53 |  |
| *HXT5* | 126.04 | 1572.17 | 3.64 | 0 | 245.91 | 3232.48 | **3.72** | 0 | Transporter |
| *HXT4* | 1889.89 | 1548.74 | -0.29 | 6.91E-74 | 83.59 | 371.03 | **2.15** | 0 |  |
| *HXT7* | 15182.23 | 7473.64 | -1.02 | 0 | 3703.70 | 7521.77 | **1.02** | 0 |  |
| *HXT3* | 1996.95 | 1619.07 | -0.30 | 1.55E-84 | 57.88 | 429.01 | **2.89** | 0 |  |
| *HXT13* | 17.49 | 80.02 | 2.19 | 1.89E-92 | 12.02 | 11.50 | **-0.06** | 0.78 |  |
| *FBA1* | 11044.66 | 12454.31 | 0.17 | 4.3E-114 | 5002.25 | 13322.56 | **1.41** | 0 | Carbon metabolism |
| *TDH1* | 12778.71 | 21534.88 | 0.75 | 0 | 9179.76 | 18373.37 | **1.00** | 0 |  |
| *TDH2* | 1465.60 | 2086.78 | 0.51 | 7.1E-134 | 567.16 | 2670.86 | **2.24** | 0 |  |
| *PGK1* | 6298.14 | 8989.43 | 0.51 | 0 | 1563.69 | 3455.05 | **1.14** | 0 |  |
| *ENO1* | 9809.89 | 16887.13 | 0.78 | 0 | 5197.43 | 8164.26 | **0.65** | 0 |  |
| *PDC1* | 1864.42 | 3142.21 | 0.75 | 0 | 362.23 | 2422.52 | **2.74** | 0 |  |
| *ADH4* | 1775.47 | 5386.14 | 1.60 | 0 | 444.40 | 1106.41 | **1.32** | 0 |  |

**Table S5 (Continued)**

| *ADH2* | 57.59 | 185.48 | 1.69 | 1.11E-91 | 330.01 | 703.13 | **1.09** | 2.8E-180 |  |
| --- | --- | --- | --- | --- | --- | --- | --- | --- | --- |
| *ALD2* | 74.73 | 122.18 | 0.71 | 1.81E-23 | 46.16 | 106.43 | **1.21** | 2.06E-48 |  |
| *ALD3* | 139.60 | 557.10 | 2.00 | 0 | 43.71 | 251.79 | **2.53** | 4E-308 |  |
| *ACS1* | 86.69 | 461.21 | 2.41 | 0 | 178.77 | 657.60 | **1.88** | 0 |  |
| *ACO1* | 196.94 | 656.40 | 1.74 | 0 | 330.84 | 1212.61 | **1.87** | 0 |  |
| *KGD1* | 138.09 | 251.04 | 0.86 | 1.6E-128 | 426.21 | 709.49 | **0.74** | 6.7E-280 |  |
| *KGD2* | 260.15 | 369.04 | 0.50 | 3.45E-34 | 534.86 | 858.93 | **0.68** | 3.7E-135 |  |
| *SDH1* | 10.89 | 26.04 | 1.26 | 9.08E-17 | 555.98 | 772.66 | **0.47** | 3.62E-89 |  |
| *MDH2* | 117.83 | 304.23 | 1.37 | 3.4E-119 | 543.00 | 1062.57 | **0.97** | 6E-243 |  |
| *GPM2* | 117.75 | 352.82 | 1.58 | 5.2E-140 | 64.49 | 379.99 | **2.56** | 3.9E-284 |  |
| *ENO2* | 1368.78 | 1105.83 | -0.31 | 2.1E-46 | 520.60 | 1281.48 | **1.30** | 0 |  |
| *TDH3* | 14229.56 | 16313.47 | 0.20 | 1.4E-175 | 3433.61 | 7578.80 | **1.14** | 0 |  |
| *MDH2* | 117.83 | 304.23 | 1.37 | 3.4E-119 | 543.00 | 1062.57 | **0.97** | 6E-243 |  |
| *PGI1* | 792.75 | 761.05 | -0.06 | 0.02 | 651.32 | 1070.24 | **0.72** | 1.3E-218 |  |
| *PCK1* | 32.85 | 32.78 | 0.00 | 0.98 | 2719.16 | 4355.12 | **0.68** | 0 |  |
| *TPI1* | 5871.88 | 6801.87 | 0.21 | 2.23E-63 | 2808.59 | 4402.73 | **0.65** | 0 |  |
| *PYC1* | 384.56 | 229.20 | -0.75 | 8.1E-178 | 912.94 | 1107.05 | **0.28** | 4.23E-88 |  |
| *PYC2* | 147.26 | 197.33 | 0.42 | 2.96E-34 | 101.35 | 114.62 | **0.18** | 2.74E-05 |  |
| *STL1* | 12.89 | 11.29 | -0.19 | 0.31 | 47.38 | 170.05 | **1.84** | 4.22E-159 |  |
| *GUT1* | 446.74 | 811.24 | 0.86 | 5.57E-285 | 576.22 | 954.21 | **0.73** | 3.11E-257 |  |
| *GUT2* | 330.65 | 442.67 | 0.42 | 3.04E-41 | 187.1 | 507.41 | **1.44** | 0 |  |
| *GRE3* | 170.63 | 763.84 | 2.16 | 0 | 112.34 | 125.69 | **0.16** | 0.03 | Xylose metabolism |
| *GCY1* | 685.01 | 1116.68 | 0.71 | 5E-120 | 434.01 | 244.73 | **-0.83** | 1.1E-61 |  |
| *YPR1* | 160.40 | 265.22 | 0.73 | 2.61E-31 | 84.79 | 142.27 | **0.75** | 8.09E-19 |  |
| *XYL2* | 97.92 | 138.26 | 0.50 | 1.27E-10 | 53.70 | 72.24 | **0.43** | 3.76E-05 |  |

**Table S5 (Continued)**

| *SOR1* | 16.03 | 8.74 | -0.88 | 0.000417 | 13.54 | 4.58 | **-1.56** | 2.19E-07 |  |
| --- | --- | --- | --- | --- | --- | --- | --- | --- | --- |
| *SOR2* | 6.41 | 0.00 | -12.65 | 4E-12 | 10.63 | 0.00 | **10.63** | 5.69E-20 |  |
| *XKS1* | 94.71 | 180.26 | 0.93 | 3.6E-62 | 28.34 | 47.91 | **0.76** | 3.03E-13 |  |
| *TKL2* | 18.69 | 292.95 | 3.97 | 0 | 22.30 | 271.05 | **3.60** | 0 |  |
| *FPS1* | 82.15 | 50.43 | -0.70 | 8.86E-21 | 123.52 | 68.09 | **-0.86** | 2.02E-42 | Xylose metabolism-related |
| *PHO13* | 112.14 | 13.17 | -3.09 | 3.4E-102 | 68.53 | 64.72 | **-0.08** | 0.50 |  |
| *CTT1* | 487.38 | 2417.92 | 2.31 | 0 | 131.32 | 1425.63 | **3.44** | 0 | Antioxidant enzymes |
| *CTA1* | 37.84 | 52.47 | 0.47 | 5.04E-06 | 90.76 | 804.05 | **3.15** | 0 |  |
| *PRX1* | 421.46 | 943.46 | 1.16 | 3.04E-193 | 621.73 | 1245.98 | **1.00** | 1.73E-205 |  |
| *HSP12* | 5208.82 | 20747.30 | 1.99 | 0 | 1479.27 | 8026.89 | **2.44** | 0 |  |
| *SOD2* | 613.96 | 1067.11 | 0.80 | 1.8E-104 | 1145.03 | 3273.72 | **1.52** | 0 |  |
| *TRX1* | 687.15 | 242.55 | -1.50 | 3.69E-74 | 253.85 | 192.04 | **-0.40** | 0.000636 |  |
| *ERG1* | 110.41 | 181.30 | 0.72 | 1.05E-33 | 72.16 | 113.64 | **0.66** | 3.96E-19 | Ergosterol biosynthesis |
| *ERG11* | 54.71 | 63.97 | 0.23 | 0.01 | 227.29 | 476.06 | **1.07** | 9.3E-182 |  |
| *CYB5* | 19.33 | 24.32 | 0.33 | 0.30 | 18.98 | 121.02 | **2.67** | 6.77E-35 |  |
| *ERG24* | 80.87 | 91.25 | 0.17 | 0.03 | 31.02 | 66.49 | **1.10** | 1.96E-23 |  |
| *ERG25* | 129.28 | 203.94 | 0.66 | 1.23E-20 | 335.81 | 602.97 | **0.84** | 2.29E-90 |  |
| *ERG26* | 111.99 | 235.18 | 1.07 | 1.25E-58 | 64.64 | 123.39 | **0.93** | 4.75E-26 |  |
| *ERG28* | 313.26 | 493.24 | 0.65 | 2.74E-22 | 85.88 | 206.11 | **1.26** | 4.54E-28 |  |
| *ERG5* | 37.70 | 49.05 | 0.38 | 0.00022 | 62.57 | 195.44 | **1.64** | 3.5E-147 |  |
| *ERG7* | 66.09 | 38.68 | -0.77 | 1.78E-21 | 26.88 | 45.43 | **0.76** | 4.09E-15 |  |
| *ERG6* | 132.07 | 80.12 | -0.72 | 1.32E-19 | 151.52 | 60.67 | **-1.32** | 3.83E-58 |  |
| *ERG3* | 133.18 | 86.40 | -0.62 | 5.89E-15 | 499.65 | 577.69 | **0.21** | 1E-09 |  |

The grey parts stand for invalid number with the P-value>0.001.

**Figure legends**

**Fig. S1 Fermentation performance of *S. cerevisiae* YB-2625 and *S. cerevisiae* S288C during mixed sugar fermentation and overview of the transcriptomic data.**

A), Growth ability of the two strains; B) and C), comparison of xylitol and ethanol production of the two strains; D), Number of different expression genes under the conditions of YBXG vs SCXG and YBX vs SCX. Batch fermentation was performed in 100 mL medium containing 4 g/L yeast extract, 3 g/L peptone, 80 g/L glucose and 20 g/L xylose in 250 mL Erlenmeyer flasks with initial OD_600_~0.2 at 150 rpm and 30°C. The results shown were the mean values of triplicate experiments.

**Fig. S2 Transcriptional regulatory network between Mig2 and other transcription factors.** Fold changes of the transcription factors in the condition of YBXG vs SCXG were presented.

**Fig. S3 Heat map of the genes regulated by *TOG1* revealed in the comparative transcriptomic analysis data.**

**Fig. S4 Effect of xylitol treatment on transcription of key genes in *S. cerevisiae* YB-2625.** Cells (OD_600_ 1.0) of *S. cerevisiae* YB-2625 were collected and transferred to YP (4 g/L yeast extract, 3 g/L peptone) medium with 10 g/L xylitol, and YP medium without addition of xylitol acted as a control. After treatment for 3 h, relative expression levels of *CTT1*, *CAT1* and *HSP12* were detected by RT-qPCR.

**B)**

**A)**

**D)**

**C)**

**Fig. S1**


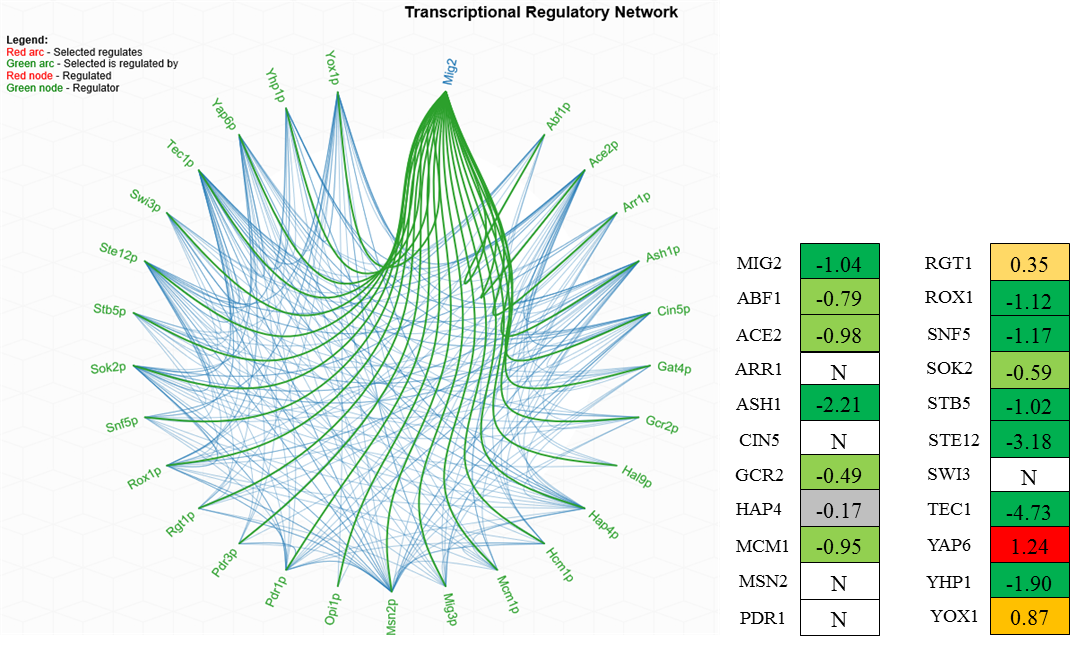


**Fig. S2**


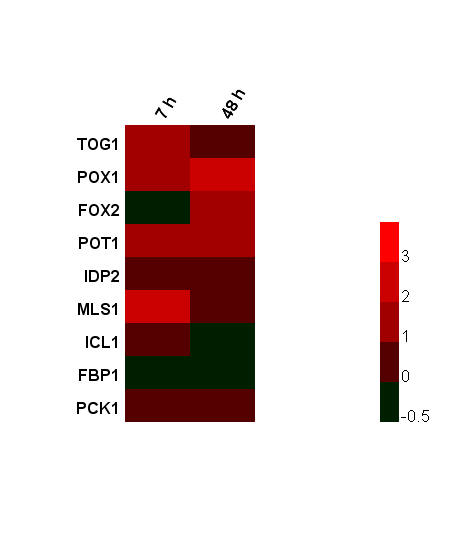

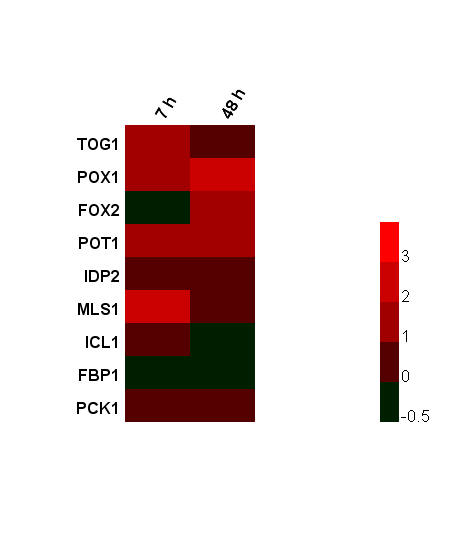


**Fig. S3**


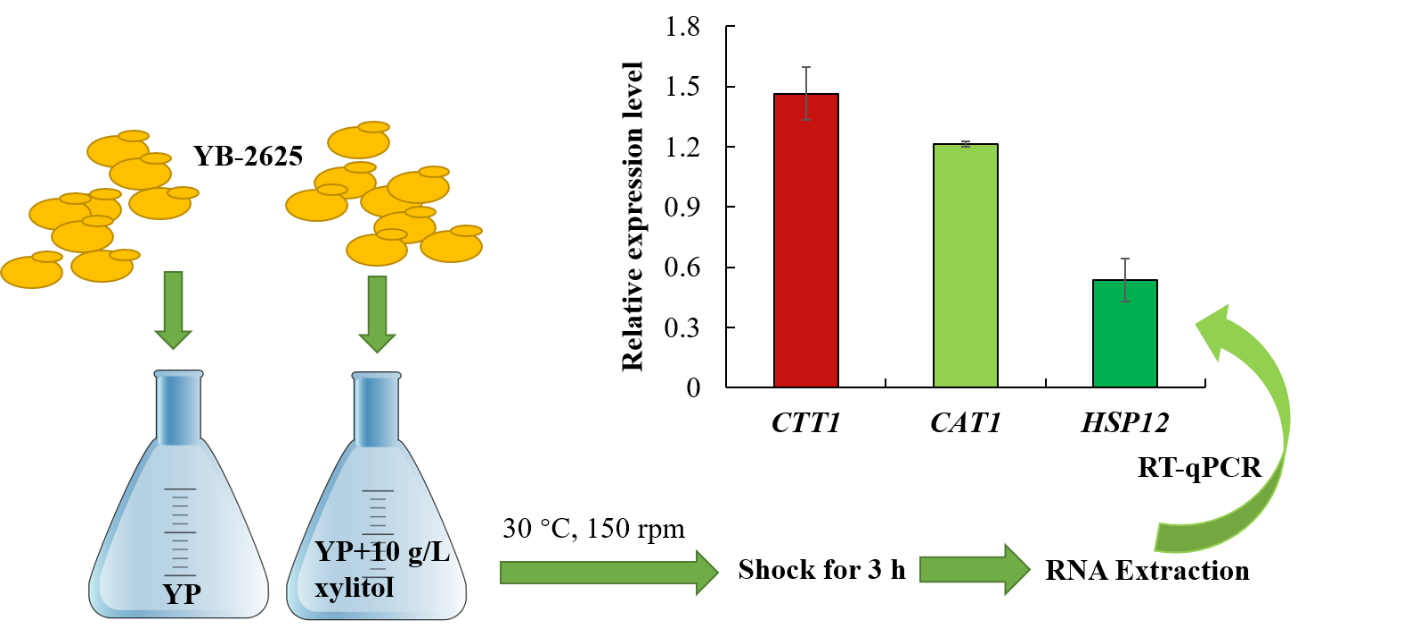


**Fig. S4**
